# Secondary metabolic profiling, antioxidant potential, enzyme inhibitory activities and in silico and ADME studies: a multifunctional approach to reveal medicinal and industrial potential of *Tanacetum falconeri*

**DOI:** 10.1186/s12906-024-04459-5

**Published:** 2024-04-22

**Authors:** Muhammad Imran Tousif, Zaheer Abbas, Mamona Nazir, Muhammad Saleem, Saba Tauseef, Areeba Hassan, Sajid Ali, Maqsood Ahmed, Jallat Khan, Gokhan Zengin, Abeer Hashem, Khalid F. Almutairi, Graciela Dolores Avila-Quezada, Elsayed Fathi Abd-Allah

**Affiliations:** 1 Department of Chemistry, Division of Science and Technology, University of Education, Lahore, Pakistan; 2 Department of Botany, Division of Science and Technology, University of Education, Lahore, Pakistan; 3 Department of Chemistry, Government Sadiq College Women University Bahawalpur, Bahawalpur, 63100 Pakistan; 4 Institute of Chemistry, Baghdad Campus, The Islamia University of Bahawalpur, Bahawalpur, 63100 Pakistan; 5 Dr. Panjwani Center for Molecular Medicine and Drug Research., International Center for Chemical and Biological Sciences, University of Karachi, Karachi, Pakistan; 6 Department of Bioinformatics, Institute of Biochemistry, Biotechnology & Bioinformatics, Baghdad Campus, The Islamia University of Bahawalpur, Bahawalpur, 63100 Pakistan; 7 Department of Information Sciences, Division of Science and Technology, University of Education Lahore, Lahore, Pakistan; 8 Department of Pharmaceutical Chemistry, Faculty of Pharmacy, The Islamia University of Bahawalpur, Bahawalpur, 63100 Pakistan; 9 Institute of Chemistry, Khwaja Fareed University of Engineering & Information Technology, Rahim Yar Khan, Pakistan; 10 Science Faculty, Department of Biology, Selcuk University, Konya, Turkey; 11 Botany and Microbiology Department, College of Science, King Saud University, P.O. Box. 2460, 11451 Riyadh, Saudi Arabia; 12 Facultad de Ciencias Agrotecnológicas, Universidad Autónoma de Chihuahua, 31350 Chihuahua, Chihuahua, México; 13 Plant Production Department, College of Food and Agricultural Sciences, King Saud University, P.O. Box. 2460, 11451 Riyadh, Saudi Arabia

**Keywords:** *Tanacetum falconeri*, Total bioactive contents; antioxidant activities, Enzyme inhibition activities, In silico studies

## Abstract

*Tanacetum falconeri* is a significant flowering plant that possesses cytotoxic, insecticidal, antibacterial, and phytotoxic properties. Its chemodiversity and bioactivities, however, have not been thoroughly investigated. In this work, several extracts from various parts of *T. falconeri* were assessed for their chemical profile, antioxidant activity, and potential for enzyme inhibition. The total phenolic contents of *T. falconeri* varied from 40.28 ± 0.47 mg GAE/g to 11.92 ± 0.22 mg GAE/g in various extracts, while flavonoid contents were found highest in TFFM (36.79 ± 0.36 mg QE/g extract) and lowest (11.08 ± 0.22 mg QE/g extract) in TFSC (chloroform extract of stem) in similar pattern as found in total phenolic contents. Highest DPPH inhibition was observed for TFFC (49.58 ± 0.11 mg TE/g extract) and TFSM (46.33 ± 0.10 mg TE/g extract), whereas, TFSM was also potentially active against (98.95 ± 0.57 mg TE/g) ABTS radical. In addition, TFSM was also most active in metal reducing assays: CUPRAC (151.76 ± 1.59 mg TE/g extract) and FRAP (101.30 ± 0.32 mg TE/g extract). In phosphomolybdenum assay, the highest activity was found for TFFE (1.71 ± 0.03 mg TE/g extract), TFSM (1.64 ± 0.035 mg TE/g extract), TFSH (1.60 ± 0.033 mg TE/g extract) and TFFH (1.58 ± 0.08 mg TE/g extract), while highest metal chelating activity was recorded for TFSH (25.93 ± 0.79 mg EDTAE/g extract), TFSE (22.90 ± 1.12 mg EDTAE/g extract) and TFSC (19.31 ± 0.50 mg EDTAE/g extract). In biological screening, all extracts had stronger inhibitory capacity against AChE while in case of BChE the chloroform extract of flower (TFFC) and stem (TFSC) showed the highest activities with inhibitory values of 2.57 ± 0.24 and 2.10 ± 0.18 respectively. Similarly, TFFC and TFSC had stronger inhibitory capacity (1.09 ± 0.015 and 1.08 ± 0.002 mmol ACAE/g extract) against α-Amylase and (0.50 ± 0.02 and 0.55 ± 0.02 mmol ACAE/g extract) α-Glucosidase. UHPLC-MS study of methanolic extract revealed the presence of 133 components including sterols, triterpenes, flavonoids, alkaloids, and coumarins. The total phenolic contents were substantially linked with all antioxidant assays in multivariate analysis. These findings were validated by docking investigations, which revealed that the selected compounds exhibited high binding free energy with the enzymes tested. Finally, it was found that *T. falconeri* is a viable industrial crop with potential use in the production of functional goods and nutraceuticals.

## Introduction

*Tanacetum falconeri* is an important flowering herb belong to plant family Asteraceae [1]. It is mostly found in the rocky talus, near the lakes, valley plains or grassy ridges in different parts of Pakistan. Locally, the powdered leaves and extract of leaves of *T. falconeri* is used against various abdominal problems, whereas, its flowers and buds are beneficial in treating asthma, jaundice and blood pressure problems [2, 3]. Different plant parts are utilised to treat joint discomfort after being dried in the shade [4]. The habitants of Kallaway Indians and the Andes mountains used these plants for back ache, abdominal pain and gastric trouble [5]. The Mexican people used it as a tonic to regulate menstruation and as an antispasmodic. In Venezuela, it's used to cure earaches [6]. A scanty work on chemodiversity and biological potential of *T. falconeri* has been reported in literature, however, other *Tanacetum* plants are rich in terpenes mostly as essential oils [7–13], sterols [14–16], phenolic acids and flavonoids [17]. Due to the presence of variety of bioactive compounds, *Tanacetum* plants extracts have shown various biological activities like anti-inflammatory, antiviral, antifungal, antibacterial and antioxidant [18], edema [19], antibacterial [14, 20], fungicidal activity [21], antioxidant [22], anti-inflammatory [23], anthelmintic, Anticoagulant and antifibrinolytic, insecticidal [14], and anti-ulcer [24, 25] and antitumor [26]. *Tanacetum* plants have also showed anti-Leishmanial*,* antibacterial [27], antimalarial [28] activities. Despite of the lack of phytochemical investigation, *Tanacetum* plants has received recognition as a potential nematocidal, insecticidal, antibacterial, cytotoxic, and phytotoxic herbs [29]. Therefore, the diverse chemical profiling of *Tanacetum* plants, and their medicinal uses prompted us to investigate *T. falconeri* for its chemodiversity and biological potential. The goal of this study was to evaluate the traditional therapeutic applications of *T. falconeri* by evaluating the various extracts for their total bioactive content, full secondary metabolic profile, and bioactivities. In vitro tests were conducted to evaluate the anti-oxidant (DPPH, ABTS, FRAP, CUPRAC, phosphomolybdenum, ferrous chelating) and enzyme inhibitory capabilities of all extracts against various enzymes linked to skin, neurodegenerative, and diabetic illnesses. Additionally, multivariate analysis and docking investigations were carried out.

## Experimental procedures

### Collection of the plant material and identification

The plant material was collected from Shigar District, Gilgit-Baltistan, Pakistan and was identified by Dr. Zaheer Abbas, a taxonomist at the University of Education, DG Khan Campus, Dera Ghazi Khan, where a voucher specimen No. BT-0063 has been deposited in the herbarium of same university.

### Preparation of the extracts

The collected plant material was divided into flower (TFF) and stem with leaves (TFS) parts, which were then dried under shade for one week. Each part (600 and 800 g, respectively) was divided into four parts, which were then extracted separately through maceration using *n*-hexane, chloroform, ethyl acetate and methanol to get crude extracts of the stem: TFSM: methanolic extract of stem; TFSH: hexane extract of stem; TFSE: ethyl acetate extract of stem; TFSC: chloroform extract of stem and flowers extracts: TFFM: methanolic extract of flowers; TFFH: hexane extract of flowers; TFFE: ethyl acetate extract of flowers; TFFC: chloroform extract of flowers. All these extracts were then studied for their phenolic and flavonoid contents, antioxidant and enzyme inhibition studies and chemodiversity.

### Estimation of Total phenolic (TPC) and Total flavonoid (TFC) contents

The Estimation of total phenolic (TPC) and total flavonoid (TFC) contents were done through same methods as we reported previously [30–33]. The results of total phenolic contents (TPC) were presented in milligrams of gallic acid equivalent per grams of extract (mg GAE/g extract). The total flavonoid contents (TFC) results were reported in milligrams of rutin equivalent per grams of extract (mg RE/g extract).

### Antioxidant activities assays

The antioxidant activities of extracts were measured by following pre-established protocols as we reported previously [30–33]. For FRAP, ABTS, DPPH, CUPRAC, and total antioxidant capacity, trolox equivalent was utilized as standard and results were expressed as mg TE/g extract; while for metal chelating assays, ethylene diamine tetraacetic acid (EDTA) was the standard and results were expressed as mg TE/g extract.

### Enzyme inhibition assays

The α-amylase, α-glucosidase, BChE, tyrosinase, and AChE enzyme inhibitory assays were conducted using previously published methods [30–33]. Acarbose (mmol ACAE/g extract) was used as a standard to measure the inhibitory activity of α-amylase and α-glucosidase. Galantamine (mg GALAE/g extract) was used to measure the inhibitory activity of AChE and BChE, and kojic acid (mmol KAE/g extract) was used to measure the inhibitory activity of tyrosinase.

### UHPLC-MS analysis

UHPLC-MS (ultra-high performance liquid chromatography mass spectrometry) analysis was used to profile secondary metabolites using an Agilent 1290 Infinity UHPLC system coupled to an Agilent 6520 Accurate-Mass Q-TOF mass spectrometer with dual ESI source, as we previously reported [30–32]. The column was an Agilent Zorbax Eclipse XDB-C18 with 3.5 m in thickness and 2.1 × 150 mm in diameter. A 0.1% formic acid solution in water served as mobile phase A, while a 0.1% formic acid solution in acetonitrile served as mobile phase B. A consistent flow rate of 0.5 millilitres per minute was maintained. One microliter of methanolic extract was given for twenty-five minutes, and then there was a five-minute post-run period. The secondary metabolites were found using the METLIN database.

### Statistical analysis

The experiments were performed in triplicate, and differences between the extracts were compared using an ANOVA and Tukey's test. Pearson correlation analysis was used to establish the link between total bioactive components and biological activity assays. Graph Pad Prism (version 9.2) was used for the analysis. To assess the degree of similarity or difference between the extracts, a PCA was carried out using SIMCA (version 14.0).

### Docking study methodology

The chemical structures of the five enzymes with the highest resolution were downloaded from the protein data bank in PDB format. Discovery Studio (DS 2021Client) software was employed to formulate protein molecules. Attached chemical moieties (water molecules and other ligand) were removed from macromolecules. Afterward, they were transferred onto the PyRx program (version 0.8) for docking purposes in pdbqt file that contains a protein structure with hydrogens in all polar residues. The structures of selected ligands were acquired from the Pubchem as 3D SDF formats. The software specification and procedure of docking were followed as described by Ahmed et al., [34]. The enzyme molecules were loaded into PyRx and converted to macromolecules by using autodock embedded in PyRx software. Then the ligands were attached using the open babel tool, and energy was minimized to obtain the stable structure; then, ligands were converted to pdbqt format. The docking site on the protein target was defined by establishing a grid box, which was maximized using “maximize” option for better coverage of active site and exhaustiveness was 8. The other settings of the software were used as “default”. The best conformation with the lowest docked energy was chosen after the docking search was completed. The molecular docking result for each compound was visualized as an output pdbqt file by using the molecular graphics laboratory (mgltool) tool. Interactions were finally visualized in discovery studio by using mgltool, to determine some specific contacts between the atoms of the test compounds and amino acids residues of the studied protein molecules [35].

## Results and discussion

### Total phenolic (TPC) and flavonoid (TFC) contents of T. falconeri

Phenolic compounds are important component of nutraceuticals and functional foods because of their antioxidant properties. The antioxidant properties of phenolics are usually attributed to the presence of hydroxyl group(s) on the benzene ring, which goes about an electron donor and consequently and straight forwardly includes in quenching free radicals. In the present study, several solvent-based crude stem and flower extracts of *T. falconeri* were screened for their total phenolic and flavonoid contents. Total phenolic contents (TPC) observed in the methanol extract of flower (TFFM) were high (40.28 ± 0.47 mg GAE/g extract), followed by the TFFH extract (33.00 ± 0.67 mg GAE/g extract). Although the same trend of TPC for stem extracts was seen in TFSM (22.21 ± 0.17 mg GAE/g extract) and TFSH (24.34 ± 0.49 mg GAE/g extract) but were lower than those of respective extracts of the flower. Ethyl acetate extracts from both the sources were next in line (Table 1). Similarly, total flavonoids contents (TFC) were also observed high in flower extracts (TFFM 36.79 ± 0.36 mg QE/g extract and TFFH 32.80 ± 0.80 mg QE/g extract), followed by stem extracts (TFSM 17.68 ± 0.32 mg QE/g extract and TFSE 23.38 ± 0.17 mg QE/g extract). In both the cases, lowest phenolic and flavonoid contents were calculated in chloroform extracts (Table 1). Usually phenolics and flavonoids are relatively polar compounds; therefore, their high concentration in methanolic or ethyl acetate extracts seems reasonable, however, in case of flower extracts (TFFH and TFSH), the high amount of phenolic contents could be attributed to the possible presence of esters of phenolic acids in the extracts. Literature reports also substantiate our findings where methanolic extracts of *Tanacetum* plants have been reported to be rich in phenolic contents [36]. Another report describes that *Tanacetum* species produce high level of vanillic acid, and caffeic acid, catechin and quercetin [37] and other phenolics and flavonoids [38, 39].; The presence of caffeoylquinic acids in *Tanacetum* species [40, 41] substantiates our deduction that phenolic acid esters are present in *T. falconeri*, which are extracted in low polar solvents, and thus TFFH and TFSH also afforded high amount of phenolic contents.

**Table 1:** Total bioactive contents of *T. falconeri*

Test Samples	TPC (mg GAE/g extract)	TFC (mg QE/g extract)
TFFM	40.28 ± 0.47^a^	36.79 ± 0.36^a^
TFFH	33.00 ± 0.67^b^	32.80 ± 0.80^b^
TFFE	17.37 ± 0.23^e^	11.65 ± 0.09^f^
TFFC	11.92 ± 0.22^f^	3.22 ± 0.05^g^
TFSM	22.21 ± 0.17^d^	17.68 ± 0.32^d^
TFSH	24.34 ± 0.49^c^	13.39 ± 0.30^e^
TFSE	22.18 ± 0.33^d^	23.38 ± 0.17^c^
TFSC	18.07 ± 0.18^e^	11.08 ± 0.22^f^

TFFM: methanolic extract of flower of *T. falconeri*; TFFH: hexane extract of flower of *T. falconeri*; TFFE: ethyl acetate extract of flower of *T. falconeri*; TFFC: chloroform extract of flower of *T. falconeri*; TFSM: methanolic extract of stem of *T. falconeri*; TFSH: hexane extract of stem of *T. falconeri*; TFSE: ethyl acetate extract of stem of *T. falconeri*; TFSC: chloroform extract of stem of *T. falconeri*. Different letters in same column indicate significant differences in the tested extracts (*p* < 0.05)

### Antioxidant activities of the extracts of T. falconeri

Research showed that the antioxidant activity of a plant extract is usually associated to the phenolic contents, i.e. higher the phenolic contents, higher will be the activity [42]. However, in the present study, the highest DPPH free radical scavenging activity (TFFM; 49.58 ± 0.11 mg TE/g extract) was associated to the methanolic flower extract, which is followed by *n*-hexane flower extract (TFFH; 47.91 ± 0.17 mg TE/g extract), whereas, methanolic stem extract (TFSM) also showed nearly similar inhibition (43.75 ± 0.41 mg TE/g extract). It is already predicted that the presence of phenolic contents in low polar solvents could be of the nature of phenolic acid esters. Literature search revealed that phenolic acid esters are potent antioxidants [43], therefore, the activity of TFFM could be attributed to such kind of compounds and other metabolites. On the other hand, the higher DPPH free radical inhibitory potential of TFSM could be attested for its high phenolic contents (Table 2). TFSM and TFFH were also significantly active, while other extracts were found inactive (Table 2). In case of ABTS free radical activity same pattern was observed as in DPPH and TFFM exhibited highest inhibition value (112.61 ± 0.15 mg TE/g extract). The next in line were TFFH, TFSM and TFSE (Table 2) with values of 84.60 ± 0.57, 73.43 ± 2.77 and 62.51 ± 0.97 respectively. In metal ion reducing power assays, again the TFFM was highly active (CUPRAC: 160.48 ± 6.59 mg TE/g extract; FRAP: 102.58 ± 2.62 mg TE/g extract), followed by the methanolic extract of stem (TFSM). All other extracts also exhibited significant and comparable metal reducing power (Table 2). TFSE was most active in phosphomolybdenum with the value of 1.71 ± 0.03 mg TE/g extract, whereas, TFFH and TFSH were also significantly active with the values of 1.64 ± 0.00 and 1.58 ± 0.08 mg TE/g extract, respectively. Highest metal chelating activity was found for stem extracts, since TFSC and TFSM displayed chelating power as 18.06 ± 0.61 and 15.57 ± 0.22 mg TE/g extract, whereas, flower extracts were found weak chelators (Table 2). It if further noticed that more polar extracts were also weak chelators, however, overall the present study revealed that *T. falconeri* is a potential antioxidant plant to be considered for its uses in health promoting formulations.

**Table 2:** Antioxidant activities of the extracts of *T. falconeri*

Test Samples	DPPH (mg TE/g extract)	ABTS (mg TE/g extract)	CUPRAC (mg TE/g extract)	FRAP (mg TE/g extract)	Phosphomolybdenum (mg TE/g extract)	metal chelating (mg EDTAE/g extract)
TFFM	49.58 ± 0.11^a^	112.61 ± 0.15^a^	160.48 ± 6.59^a^	102.58 ± 2.62^a^	1.12 ± 0.01^ cd^	13.02 ± 0.39^c^
TFFH	47.91 ± 0.17^b^	84.60 ± 0.57^b^	123.27 ± 2.38^b^	67.17 ± 0.21^b^	1.64 ± 0.00^a^	10.05 ± 0.60^d^
TFFE	5.87 ± 0.45^f^	36.60 ± 1.15^f^	49.58 ± 2.90^d^	17.93 ± 0.05^f^	1.25 ± 0.02^bc^	8.73 ± 0.29^d^
TFFC	Not active	15.95 ± 1.20^ g^	35.17 ± 0.94^e^	15.23 ± 0.48^f^	1.00 ± 0.02^d^	14.03 ± 0.28^bc^
TFSM	43.75 ± 0.41^c^	73.43 ± 2.77^c^	82.73 ± 1.59^c^	50.64 ± 0.22^c^	1.10 ± 0.02^d^	15.57 ± 0.22^b^
TFSH	19.20 ± 0.37^e^	47.23 ± 1.11^e^	77.22 ± 1.98^c^	40.40 ± 1.36^d^	1.58 ± 0.08^a^	9.31 ± 0.70^d^
TFSE	22.42 ± 0.38^d^	62.51 ± 0.97^d^	77.13 ± 0.88^c^	50.20 ± 0.82^c^	1.71 ± 0.03^a^	9.04 ± 0.69^d^
TFSC	5.95 ± 0.61^f^	38.33 ± 2.11^f^	51.75 ± 0.52^d^	23.93 ± 0.13^e^	1.36 ± 0.03^b^	18.06 ± 0.61^a^

TFFM: methanolic extract of flower of *T. falconeri*; TFFH: hexane extract of flower of *T. falconeri*; TFFE: ethyl acetate extract of flower of *T. falconeri*; TFFC: chloroform extract of flower of *T. falconeri*; TFSM: methanolic extract of stem of *T. falconeri*; TFSH: hexane extract of stem of *T. falconeri*; TFSE: ethyl acetate extract of stem of *T. falconeri*; TFSC: methanolic extract of stem of *T. falconeri*. Different letters in same column indicate significant differences in the tested extracts (*p* < 0.05)

### Enzyme inhibition activities of the extracts of T. falconeri

#### AChE and BChE enzyme inhibition activities

Alzheimer's disease (AD), a noncommunicable disease (NCDs) has been identified as a largely increasing health challenge worldwide. It is an irreversible, progressive form of dementia, associated with an ongoing decline of brain functioning [44] and thus causes memory loss. The World Health Organization (WHO) has reported that more than 30 million people are afflicted by AD and this number is expected to become double every two decades to reach ~ 115 million by 2050. This problem is thus expected to weaken the social and economic development and may affect the social services [45]. Acetylcholine (ACh) and buytrylcholine (BCh) are important neurotransmitters requires for proper brain, memory and body functioning Therefore, low levels of cholines lead to memory issues and muscle disorders. Acetylcholinesterase (AChE) and butyrylcholinesterase (BChE) are the enzymes that hydrolyse acetycholine and butyrylcholine, respectively [46]. The inhibitors of these two enzymes results into accumulation of the neurotransmitter acetylcholine and enhanced stimulation of postsynaptic cholinergic receptors [47, 48]. Natural products have already proven to be promising sources of useful acetylcholinesterase (AChE) inhibitors [49]. The currently approved drugs for AD, galantamine and rivastigmine, are plant-derived alkaloids, which offer symptomatic relief from AD [50]. These facts suggest to investigate the use of medicinal plants and their formulations to prevent and treat neurodegenerative disease [51].

In this study, all the extracts of *T. falconeri* were evaluated against AChE and BChE enzymes. Methanolic (TFFM) and ethyl acetate (TFFE) extracts f flowers, while ethyl acetate (TFSE) and chloroform (TFSC) extracts of stem were most but equally active with inhibitory values as 4.09 ± 0.09, 4.53 ± 0.13, 4.00 ± 0.23 and 4.03 ± 0.22 mg GALAE/g extract, respectively, against AChE, whereas, TFFE, TFFC and TFSC were most active against BChE with values as 2.09 ± 0.18, 2.57 ± 0.24 and 2.10 ± 0.18 mg GALAE/g extract. These observations revealed that ethyl acetate and chloroform extracts are more active against these enzymes.

#### Tyrosinase enzyme inhibition activities

Browning of raw food and hyperpigmentation of human skin are two undesirable processes caused a group of copper-containing enzyme tyrosinase (EC 1.14. 18.1). Hyper activity of tyrosinase causes results in a less attractive appearance and loss in nutritional quality of food and blackening of human skin [52]. Further, over production of melanin in human skin causes several skin disorders such as melasma, senile lentigines and freckles and thus exert a considerable psychosomatic effect on affected patients [53]. These problems can be controlled by using tyrosinase inhibitors. Presently available tyrosinase inhibitors like hydroquinone, arbutin, kojic acid, ascorbic acid, ellagic acid and others have different problems either in their use or the bioavailability [54].Therefore, there is a great need to discover and develop new but safer tyrosinase inhibitors. For his purpose, the medicinal plant extracts are the main agents being researched and used as tyrosinase inhibitors in these days. In the present work various extracts of T. falconeri were evaluated for their tyrosinase inhibitory activity. Methanolic extracts of both flowers (TFFM) and stem TFSM) of *T. falconeri* were the most active with inhibition values as 35.53 ± 0.35 and 35.30 ± 0.70 mg KAE/g extract, respectively followed by the hexane extracts (29.96 ± 0.10 and 32.41 ± 1.91 mg KAE/g extract, respectively. All other extracts exhibited equal but significant inhibitory potential (Table 3), which disclosed that *T. falconeri* can be a potential ingredient in cosmetic and food industry.

**Table 3:** Enzyme inhibition activities of the extracts of *T. falconeri*

Test Samples	AChE (mg GALAE/g extract)	BChE (mg GALAE/g extract)	Tyrosinase (mg KAE/g extract)	α-Amylase (mg ACAE/g extract)	α-Glucosidase (mg ACAE/g extract)
TFFM	4.09 ± 0.09^ab^	0.47 ± 0.08^cd^	35.53 ± 0.35^a^	0.38 ± 0.009^c^	0.46 ± 0.002^d^
TFFH	3.40 ± 0.28^ cd^	0.38 ± 0.06^d^	29.96 ± 0.10^bc^	0.48 ± 0.02^b^	0.86 ± 0.001^c^
TFFE	4.53 ± 0.13^a^	2.09 ± 0.18^a^	27.00 ± 0.90^c^	0.55 ± 0.02^ab^	1.06 ± 0.002^a^
TFFC	4.14 ± 0.08^ab^	2.57 ± 0.24^a^	27.25 ± 1.29^c^	0.50 ± 0.02^ab^	1.09 ± 0.015^a^
TFSM	3.24 ± 0.12^d^	0.94 ± 0.09^bc^	35.30 ± 0.70^a^	0.41 ± 0.01^c^	0.91 ± 0.003^bc^
TFSH	3.67 ± 0.12^bcd^	1.46 ± 0.10^b^	32.4 1 ± 1.91^ab^	0.53 ± 0.01^ab^	0.87 ± 0.015^c^
TFSE	4.00 ± 0.23^abc^	1.16 ± 0.17^b^	26.79 ± 0.63^c^	0.53 ± 0.01^ab^	1.02 ± 0.107^ab^
TFSC	4.03 ± 0.22^ab^	2.10 ± 0.18^a^	27.50 ± 1.50^c^	0.55 ± 0.02^a^	1.08 ± 0.002^a^

TFFM: methanolic extract of flower of *T. falconeri*; TFFH: hexane extract of flower of *T. falconeri*; TFFE: ethyl acetate extract of flower of *T. falconeri*; TFFC: chloroform extract of flower of *T. falconeri*; TFSM: methanolic extract of stem of *T. falconeri*; TFSH: hexane extract of stem of *T. falconeri*; TFSE: ethyl acetate extract of stem of *T. falconeri*; TFSC: methanolic extract of stem of *T. falconeri.* Different letters in same column indicate significant differences in the tested extracts (*p* < 0.05)

#### α-Amylase and α-glucosidase enzyme inhibition activities

Diabetes mellitus is another major non-communicable metabolic disease that has high impact on health and economy. A published report revealed that only in 2014, 4.9 million deaths were recorded due to diabetes [55]. In diabetic patients usually the blood glucose level increases after taking meal and thus causes postprandial hyperglycemia [56]. The glycosidic linkage in carbohydrates is broken by α-amylase to produce oligosaccharides, which are then degraded to glucose by α-glucosidase [57]. Since both the enzymes digest the carbohydrates and cause diabetes [55]; inhibition of the activity of these enzymes can delay the increase in blood glucose level and reduce the risk of developing diabetes [58]. Among current inhibitors, only acarbose inhibits both α-amylase and α-glucosidase, whereas, miglitol and voglibose inhibit only α-glucosidase [59, 60]. Literature search revealed that some of the plant extracts or pure phytochemicals were found effective against both enzymes [61–64], which leads to conclude that medicinal plants can serve as potential antidiabetic agents. Therefore, in the present study, the flower and stem extracts of *T. falconeri* were evaluated for their inhibitory potential against α-amylase and α-glucosidase. Against amylase, all the extracts exhibited significant activity with inhibitory values in the range of 0.38 ± 0.009 to 0.55 ± 0.002 mg ACAE/g extract, while against α-glucosidase the inhibitory values were observed between 0.46 ± 0.002 to 1.09 ± 0.015 mg ACAE/g extract, with lowest potential in both the cases was observed for methanolic extracts (Table 3). It is reported that the plant extracts exhibit antidiabetic properties due to the combined effect of biologically active compounds like polyphenols, carotenoids, lignans, coumarins, glucosinolates, etc. [65]. These plant metabolites as a combined effect, improve the performance of pancreatic tissue by increasing insulin secretions or by reducing the intestinal absorption of glucose [66]. Therefore, the anti-amylase and anti-glucosidase activities of the extracts of *T. falconeri* could be attributed to the presence of such metabolites. Literature reports revealed that most of the plant extracts and pure compounds exhibit selective inhibition against either α-amylase or α-glucosidase; while only fewer have been found active against both the enzymes [55]. Overall the crude extracts of *T. falconeri* were found significantly active against both the enzymes; therefore, it can be a potential component of crude antidiabetic drugs.

### UHPLC-MS Analysis

**Fig. 1:**
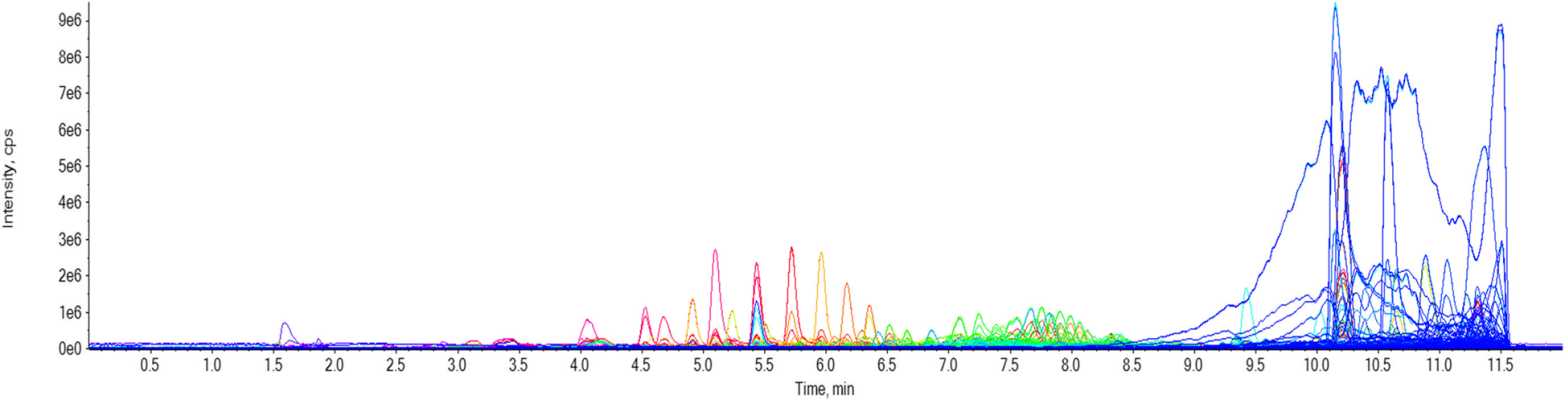
Total ion chromatogram of UHPLC-MS of methnolic extract of *T. falconeri*

UHPLC-MS Analysis of methanolic extract (Fig. 1) of flowers result in identification of 133 compounds (Table 4) of various secondary metabolites class mainly phenolic, flavonoids, Alkaloids, terpenoids and steroids. The presence of important phenolic and flavonoids compounds like 6-Caffeoylsucrose, 3-O-Feruloylquinic acid, Brosimacutin C, Quinic acid, Rutacultin, Castamollissin, Kaempferol 3-p-coumarate and Methylsyringin may be responsible for antioxidant activities [67]. These results demonstrated that *T. falconeri* is not limited to a specific class of secondary metabolites and can create a broad variety of compounds. Chemodiversity makes *T. falconeri* a valuable herb with a broad range of bioactivities.

**Table 4:** UHPLC-MS analysis of methanolic extract of flower of *T. falconeri*

Sr. No	Analyte Peak Name	Retention Time	Area / Height	Molecular formula	Compound name	Mass	Class of compound
1	381.0730 (M + K^+^)	1.51	3.93	C_19_H_18_O_6_	1,5,8-Trihydroxy-3-methyl-_2_-prenylxanthone	342.3	Xanthone
2	317.0928	1.48	4.01	C_13_H_16_O_9_	Ginnalin B	316.0794	Phenolic acid
3	429.2146	1.49	4.97	C_23_H_28_N_2_O_6_	Carapanaubine	428.1947	Indolizine
4	319.0642	1.50	4.77	C_18_H_10_N_2_O_4_	Prekinamycin	318.0641	Alkaloids
5	439.1663	1.51	3.80	C_21_H_26_O_10_	sec-o-Glucosylhamaudol	438.1526	Chromene
6	321.0363	1.50	3.45	C_18_H_8_O_6_	Erosnin	320.0321	Coumestan
7	295.0925	1.56	3.65	C_18_H_14_O_4_	7-Acetyloxy-2-methylisoflavone	294.0892	Isoflavone
8	367.1182	1.66	3.96	C_21_H_18_O_6_	Isoglycyrol	366.1103	Phyto
9	353.0613	1.70	3.64	C_19_H_12_O_7_	Daphnoretin	352.0583	Coumarin
10	685.2285	1.73	4.65	C_37_H_36_N_2_O_11_	Citbismine C	684.2319	Acridine
11	684.2245 (M + NH4^+^)	1.73	4.39	C_30_H_34_O_17_	Peonidin acetyl 3,5-diglucoside	666.1796	Anthocyanidine glycoside
12	505.1489	1.74	4.67	C_21_H_28_O_14_	6-Caffeoylsucrose	504.1479	Cinnamic acid glycoside
13	244.0595	1.77	3.26	C_13_H_9_NO_4_	Maculine	243.0532	Phyto
14	458.1606	1.81	4.17	C_20_H_27_NO_11_	Amygdalin	457.1584	Cyanogen glycoside
15	367.0863	1.82	5.49	C_20_H_14_O_7_	12a-Methoxydolineone	366.0740	Rotenoid flavonoid
16	306.1160	2.32	6.76	C_19_H_15_NO_3_	N-Acetyldehydroanonaine	305.1052	Isoquinoline alkaloid
17	431.1402	2.45	4.00	C_22_H_22_O_9_	Catechin 3-O-(1,6-dihydroxy-2-cyclohexene-1-carboxylate)	430.1264	Flavonoid
18	419.1085	2.49	2.96	C_20_H_18_O_10_	Kaempferol 3-xyloside	418.0900	Falvonoid
19	312.1150	2.75	8.56	C_14_H_17_NO_7_	Zierin	311.1005	Cyanogen glycoside
20	345.1150	3.03	7.13	C_22_H_16_O_4_	Sanaganone	344.1049	Extended flavonoid
21	378.1217 (M + Na^+^)	3.14	8.18	C_20_H_21_NO_5_	Promucosine	355.1420	Alkaloid
22	307.1367	3.57	3.03	C_20_H_18_O_3_	Lonchocarpin	306.1256	Chalcone
23	203.0709	3.70	13.48	C_12_H_10_O_3_	5-Hydroxy-2,3-dimethyl-1,4-naphthoquinone	202.0630	Naphthaquinone
24	397.1801	3.66	5.28	C_20_H_28_O_8_	4,5-Dihydroniveusin A	396.1784	Germacranolide
25	305.0988	3.76	9.90	C_16_H_16_O_6_	4'-O-Methylcatechin	304.0947	Catechin
26	329.0808	3.87	5.95	C_14_H_16_O_9_	BERGENIN	328.0794	Trihydroxybenzoic acid derivative
27	270.0759	3.87	5.71	C_15_H_11_NO_4_	Evoxanthidine	269.0688	Acridine
28	319.1141 (M + Na^+^)	4.06	8.67	C_17_H_18_O_6_	Bryaflavan	318.1103	Isoflavane
29	279.1226	4.06	7.83	C_15_H_18_O_5_	Artecanin	278.1154	Sesquiterpene lactone
30	372.1787 (M + NH4^+^)	4.36	8.11	C_21_H_22_O_5_	Mundulea flavanone B	354.1467	Flavanone
31	303.0833	4.26	7.97	C_16_H_14_O_6_	Alysifolinone	302.0790	Flavanone
32	293.1148	4.38	6.39	C_19_H_16_O_3_	Purpuritenin A	292.1099	Chalcone
33	325.1269 (M + CH3OH + H^+^)	4.37	9.25	C_15_H_16_O_6_	Dihydromikanolide	292.0947	Lactone
34	372.1803 (M + NH4^+^)	4.36	7.46	C_21_H_22_O_5_	Flemistrictin D	354.1467	Chalcone
35	369.1127	4.54	7.78	C_17_H_20_O_9_	3-O-Feruloylquinic acid	368.1107	Quinic acid derivative
36	360.1805 (M + CH3OH + H^+^)	4.54	5.57	C_19_H_21_NO_4_	Norcorydine	327.1471	Alkaloid
37	323.1477	4.52	6.18	C_17_H_22_O_6_	Tetraneurin A	322.1416	Sesquiterpene lactone
38	360.1891 (M + NH4^+^)	4.53	4.99	C_20_H_22_O_5_	Brosimacutin C	342.1467	Flavanone
39	239.1290	4.68	6.88	C_13_H_18_O_4_	1-(3-Ethyl-2,4-dihydroxy-6-methoxyphenyl)-1-butanone	238.1205	Aromatic ketone
40	416.1874 (M + NH4^+^)	4.75	6.14	C_19_H_26_O_9_	Methyl 3,4-dihydroxy-5-prenylbenzoate 3-glucoside	398.1577	Tannin
41	369.1516	4.84	6.55	C_18_H_24_O_8_	4-Hydroxy-3-prenylbenzoic acid glucoside	368.1471	Phenolic glycoside
42	281.1386	4.83	5.90	C_15_H_20_O_5_	8-Deoxy-11,13-dihydroxygrosheimin	280.1311	Sesquiterpene lactone
43	332.1685 (M + NH_4_^+^)	4.83	5.55	C_18_H_20_NO_4_	Litcubinine	314.1392	Alkaloids
44	279.1227	4.88	5.27	C_15_H_18_O_5_	Artecanin (Tanacetum parthenium)	278.1154	Sesquiterpene lactone
45	191.0735	4.89	5.74	C_11_H_10_O_3_	7-Hydroxy-2,5-dimethyl-4H-1-benzopyran-4-one	190.0630	Coumarin
46	332.1687	4.83	5.56	C_22_H_21_NO_2_	Melochinone	331.1572	Quinoline
47	444.1823	5.04	9.40	C_23_H_29_N_3_O_2_S_2_	Thiothixene	443.1701	Thioxanthene
48	404.2139 (M + CH3OH + H^+^)	4.91	5.32	C_21_H_25_NO_5_	Capaurine	371.1733	Alkaloid
49	549.2671	5.05	4.12	C_29_H_40_O_10_	Archangelolide	548.2621	Sesquiterpene lactone
50	533.2355	5.03	4.87	C_28_H_36_O_10_	Nomilinic acid	532.2308	Steroidal lactone
51	382.1971	5.05	4.80	C_19_H_27_NO_7_	Petasitenine	381.1788	Spiro epoxide
52	285.1748	5.10	5.18	C_18_H_22_NO_2_	6,7-Dihydro-4-(hydroxymethyl)-2-(p-hydroxyphenethyl)-7-methyl-5H-2-pyrindinium	284.1651	Phenol
53	283.1690	5.10	6.42	C_19_H_22_O_2_	Miltirone	282.1620	Diterpenoid
54	461.2160	5.08	6.37	C_25_H_32_O_8_	Aspidin	460.2097	Phloroglucinol
55	285.1595 (M + NH_4_^+^)	5.10	5.38	C_17_H_17_NO_2_	Assoanine	267.1259	Phenanthridine
56	251.1291	5.14	5.79	C_14_H_18_O_4_	Helinorbisabone	250.1205	Terpenoids
57	285.1329	5.14	5.55	C_14_H_20_O_6_	2-Phenylethyl beta-D-glucopyranoside	284.1260	Glycoside
58	193.0680	5.21	3.75	C_7_H_12_O_6_	Quinic acid	192.0634	Cyclitol carboxylic acid
59	452.2076	5.28	7.11	C_26_H_29_NO_6_	Piscerythramine	451.1995	Flavonoids
60	335.1238	5.36	4.55	C_21_H_18_O_4_	Calopogoniumisoflavone A	334.1205	Flavonoids
61	404.2241 (M + NH_4_^+^)	5.33	5.30	C_19_H_30_O_8_	Citroside A	386.1941	Glycoside
62	331.1079	5.38	5.34	C_18_H_18_O_6_	7-Hydroxy-5,8,2'-trimethoxyflavanone	330.1103	Flavonoids
63	267.1232	5.37	4.96	C_14_H_18_O_5_	Sapidolide A	266.1154	Lactone
64	317.0991 (M + CH3OH + H^+^)	5.37	10.44	C_16_H_12_O_5_	6-Methylapigenin	284.0685	Flavonoids
65	346.2001 (M + NH_4_^+^)	5.48	7.36	C_20_H_24_O_4_	Sclareapinone	328.1675	Quinone
66	411.1374	5.52	4.10	C_23_H_22_O_7_	Pongapinone A	410.1366	Coumarin
67	367.1725	5.55	4.93	C_19_H_26_O_7_	Orizabin	366.1679	Terpenoids
68	575.1614	5.46	1.36	C_35_H_26_O_8_	Viniferal	574.1628	Benzofuran
69	323.1089	5.67	5.72	C_19_H_16_NO_4_	Berberrubine	322.1079	Alkaloids
70	372.1986 (M + NH_4_^+^)	5.68	9.08	C_23_H_27_NO_2_	Murrayazolinine	349.2042	Alkaloids
71	374.1769 (M + NH_4_^+^)	5.75	9.07	C_13_H_24_O_11_	Galactopinitol A	356.1319	Glycoside
72	519.2568	5.74	9.43	C_24_H_38_O_12_	Cinnamoside	518.2363	Phenolic amide
73	535.2872	5.75	4.63	C_29_H_42_O_9_	Corchoroside A	534.2829	Cardenolide glycoside
74	418.2217 (M + CH3OH + H^+^)	5.87	7.80	C_22_H_27_NO_5_	O-Methylandrocymbine	385.1889	Alkaloids
75	335.1238	5.83	5.35	C_16_H_18_N_2_O_6_	Cappariloside A	334.1165	Indoles
76	561.1657	5.85	7.37	C_27_H_28_O_13_	3'-Deoxymaysin	560.1530	Flavonoids
77	408.2128	5.97	5.41	C_25_H_29_NO_4_	Ancistrocladine	407.2097	Nephthalenes
78	275.1255	5.89	9.18	C_16_H_18_O_4_	Rutacultin	274.1205	Coumarin
79	282.1697 (M + NH_4_^+^)	5.89	5.37	C_15_H_20_O_4_	Sequiterpene Lactone 326	264.1362	Terpenoids
80	353.1920	5.91	3.92	C_21_H_24_N_2_O_3_	Vobasine	352.1787	Alkaloids
81	563.1818	5.99	10.84	C_27_H_30_O_13_	Rhamnellaflavoside A	562.1686	Flavanoid
82	369.1882	5.93	4.82	C_21_H_24_N_2_O_4_	Baloxine	368.1736	Alkaloid ester
83	469.1053	6.12	2.62	C_20_H_20_O_13_	Castamollissin	468.0904	Phenolic
84	439.2165	6.10	4.62	C_26_H_30_O_6_	Kanzonol G	438.2042	Flavonoids
85	318.0725	6.11	4.48	C_19_H_11_NO_4_	Lettowianthine	317.0688	Alkaloids
86	606.3224	6.16	5.56	C_33_H_43_N_5_O_6_	Amphibine H	605.3213	Peptide
87	368.1673	6.16	6.28	C_18_H_25_NO_7_	Isatidine	367.1631	Alkaloids
88	342.1887 (M + NH_4_^+^)	6.35	6.34	C_17_H_24_O_6_	Chamissonolide	324.1573	Terpenoids
89	293.0782	6.19	4.71	C_18_H_12_O_4_	Karanjin	292.0736	Flavonoids
90	507.3096	6.30	4.33	C_29_H_38_N_4_O_4_	Mucronine A	506.2893	Peptide
91	293.1751	6.42	4.04	C_17_H_24_O_4_	9-Acetoxyfukinanolide	292.1675	Terpenoids
92	365.1555 (M + CH3OH + H^+^)	6.45	13.62	C_18_H_20_O_6_	3,3'-Dihydroxy-4',5,7-trimethoxyflavan	332.1260	Flavonoids
93	375.1748 (M + Na^+^)	6.45	7.96	C_21_H_24_N_2_O_3_	Ajmalicine	352.1787	Alkaloid
94	551.2326	6.51	3.47	C_31_H_34_O_9_	Lappaol B	550.2203	Phenylpropanoids
95	369.2056	6.60	10.05	C_23_H_28_O_4_	Quercetol B	368.1988	Phenylpropanoids
96	365.1331 (M + K^+^)	6.64	9.65	C_19_H_22_N_2_O_3_	Alkaloid AQC2	326.1630	Alkaloids
97	369.1878	6.59	8.25	C_21_H_24_N_2_O_4_	Uncarine A	368.1736	Alkaloids
98	315.1191	6.67	5.06	C_18_H_18_O_5_	Matteucinol	314.1154	Flavonoids
99	597.2793 (M + Na^+^)	6.69	5.96	C_31_H_42_O_10_	Asclepin	574.2778	Cardenolide glycoside
100	405.1265 (M + K^+^)	6.65	5.97	C_21_H_22_N_2_O_4_	Apodine	366.1580	Alkaloids
101	402.2446 (M + NH_4_^+^)	6.75	9.74	C_20_H_32_O_7_	Cinnzeylanol	384.2148	Terpenoids
102	455.0687 (M + Na^+^)	6.79	4.80	C_24_H_16_O_8_	Kaempferol 3-*p*-coumarate	432.0845	Phenolics
103	399.2339	6.85	4.83	C_23_H_30_N_2_O_4_	Desacetoxyvindoline	398.2206	Alkaloids
104	446.2690 (M + NH_4_^+^)	6.93	7.66	C_24_H_32_N_2_O_5_	Aspidoalbine	428.2311	Alkaloids
105	371.2226 (M + CH3OH + H^+^)	6.92	5.00	C_22_H_26_O_3_	5,7-Dimethoxy-8-prenylflavan	338.1882	Flavonoids
106	384.1988 (M + NH_4_^+^)	6.98	10.99	C_19_H_26_O_7_	Orizabin	366.1679	Terpenoids
107	435.1731	8.36	5.91	C_26_H_26_O_6_	Cycloartocarpin A	434.1729	Flavonoids
108	309.1449	8.49	8.04	C_20_H_20_O_3_	Isocordoin	308.1412	Phenolics
109	647.3895	8.74	11.05	C_36_H_54_O_10_	Gypsogenin 3-O-b-D-glucuronide	646.3717	Terpenoids
110	354.1438 (M + Na^+^)	8.96	5.61	C_22_H_21_NO_2_	Melochinone	331.1572	Alkaloids
111	301.0489	9.00	5.91	C_12_H_12_O_9_	Mumefural	300.0481	Furans
112	423.2096	9.20	6.47	C_26_H_30_O_5_	Alopecurone G	422.2093	Flavonoids
113	415.1712	9.27	5.81	C_23_H_26_O_7_	Heteroflavanone C	414.1679	Flavonoids
114	686.3632	9.39	9.61	C_36_H_51_N_3_O_10_	Avadharidine	685.3574	Terpenoids
115	415.2072	9.47	5.83	C_24_H_30_O_6_	Armillaripin	414.2042	Terpenoids
116	387.1727	9.63	6.36	C_18_H_26_O_9_	Methylsyringin	386.1577	Terpenoids
117	409.1384 (M + Na^+^)	9.63	7.39	C_25_H_22_O_4_	Fulvinervin B	386.1518	Flavonoids
118	229.1800	10.40	7.86	C_13_H_24_O_3_	Menthone 1,2-glyceryl ketal	228.1725	Monoterpenoid
119	289.1581	10.51	7.16	C_16_H_20_N_2_O_3_	( ±)-Rollipyrrole	288.1474	Alkaloids
120	423.2464	10.56	6.34	C_23_H_34_O_7_	Picrasin C	422.2305	Terpenoids
121	281.2062	10.56	4.42	C_19_H_24_N_2_	N-Methylaspidospermatidine	280.1939	Alkaloids
122	512.3634 (M + NH_4_^+^)	10.67	9.87	C_33_H_42_N_4_	Auricularine	494.3409	Alkaloids
123	389.1716	10.62	7.30	C_25_H_24_O_4_	Kanzonol E	388.1675	Flavonoids
124	531.3973 (M + NH_4_^+^)	10.80	9.25	C_18_H_23_NO_4_	Pandamarilactonine A	317.1627	Alkaloids
125	322.2130	10.78	6.69	C_22_H_27_NO	Phenazocine	321.2093	Alkaloids
126	368.2188 (M + NH_4_^+^)	10.89	5.09	C_16_H_30_O_6_	L-Citronellol glucoside	318.2042	Terpenoids
127	673.6284	10.96	14.58	C_23_H_37_NO_5_	Cammaconine	407.2672	Terpenoids
128	397.2128	11.06	4.91	C_23_H_28_N_2_O_4_	Echitovenine	396.2049	Alkaloids
129	600.4545 (M + NH_4_^+^)	11.22	10.79	C_37_H_58_O_5_	Hericene B	582.4284	Terpenoids
130	277.1950	11.27	5.58	C_16_H_24_N_2_O_2_	Carolinianine	276.1838	Alkaloids
131	694.3685 (M + NH_4_^+^)	11.29	5.20	C_36_H_52_O_12_	Cucurbitacin I 2-glucoside	676.3459	Terpenoids
132	275.2372	11.44	8.66	C_19_H_30_O	4,5-(methanoxy-2-methylethano)isolongifol-4-ene	274.2297	Sesquiterpenoid
133	513.3911	11.29	9.54	C_33_H_52_O_4_	Methyl 3b-hydroxy-13(18)-oleanen-28-oate	512.3866	Triterpenoid

### Data analysis

**Fig. 2:**
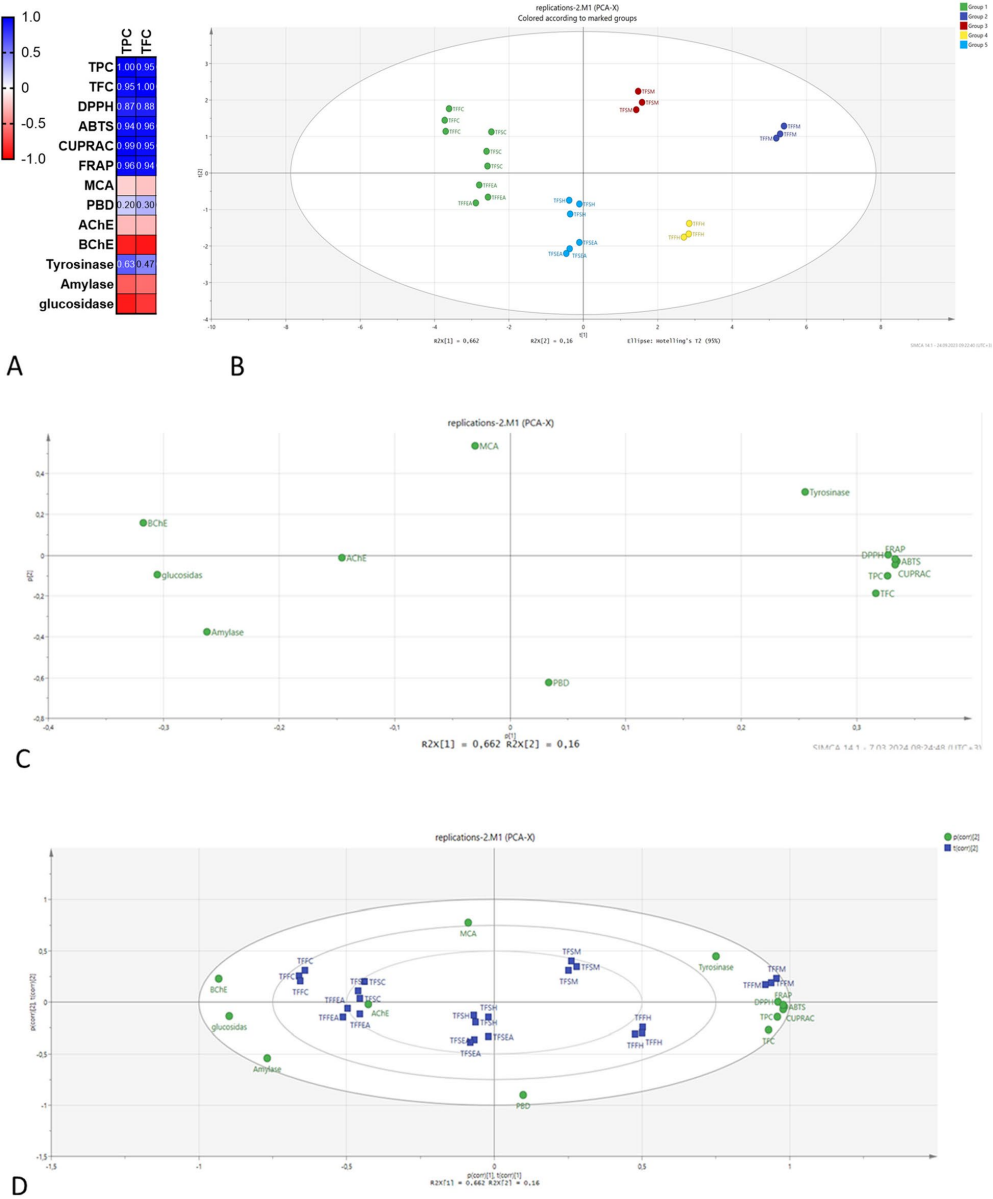
(**A**) Pearson correlation values between biological activity assays (*p* < 0.05). TPC: Total phenolic content; TFC: Total flavonoid content; ABTS: 2,2’-azino-bis(3-ethylbenzothiazoline-6-sulphonic acid; DPPH: 1,1-diphenyl-2-picrylhydrazyl; CUPRAC: Cupric reducing antioxidant capacity; FRAP: Ferric reducing antioxidant power; AChE: acetylcholinesterase; BChE: butyrylcholinesterase. **B** A plot presentation of Principal component analysis between tested samples. **C** Loading scatter plot for variables. **D** Biplot presentation between variables and tested extracts. TFFM: methanolic extract of flower of *T. falconeri*; TFFH: hexane extract of flower of *T. falconeri*; TFFE: ethyl acetate extract of flower of *T. falconeri*; TFFC: chloroform extract of flower of *T. falconeri*; TFSM: methanolic extract of stem of *T. falconeri*; TFSH: hexane extract of stem of *T. falconeri*; TFSE: ethyl acetate extract of stem of *T. falconeri*; TFSC: methanolic extract of stem of *T. falconeri*

Multivariate analysis provides a bridge between diverse parameters and their interactions. This makes it a fundamental tool in phytochemical studies to gain more information on the relationship between the chemical components and biological activities of plant extracts. For this purpose, we conducted a multivariate analysis of the extracts tested. Initially, we assessed the correlation between the total bioactive compounds and the biological activities. As illustrated in Fig. 2A, the radical quenching and reducing potentials were strongly associated with these compounds. However, metal chelation and phosphomolybdenum capacities had no association with the total of phenolic and flavonoid components. This can be elucidated by the presence of non-phenolic substances like terpenoids or peptides. In agreement with our findings, several researchers highlighted a signficant relationship between the total bioactive constituents and antioxidant properties [68, 69]. In terms of enzyme inhibitory characteristics, no relationship was found with the total bioactive components. Principal component analysis was employed to demonstrate the similarity/dissimilarity among the tested samples and R^2^ and O^2^ that shows the fitness and predictive ability of the model were found as 0.98 and 0.82, respectively (Fig. 2B). In Fig. 2C, we observed a loading scatter plot of the tested variables, and the total bioactive components and antioxidant properties were the same in the plot. However, the enzyme inhibitory effects were classified in another plot. According to Fig. 2B and 2D, the tested extracts were classified into five groups. In comparison to the other extracts, the methanol extracts from both parts showed significantly stronger antioxidant activity, thereby setting them apart from the other extracts. At the same time, the chloroform extracts had a greater enzyme inhibition effect, thus leading them to be classified in the same group. It is clear that the plant parts and extraction solvents used influenced the distribution of extracts. Our findings can be utilized for further applications involving *T. falconeri*.

### Post dock analysis

**Fig. 3:**
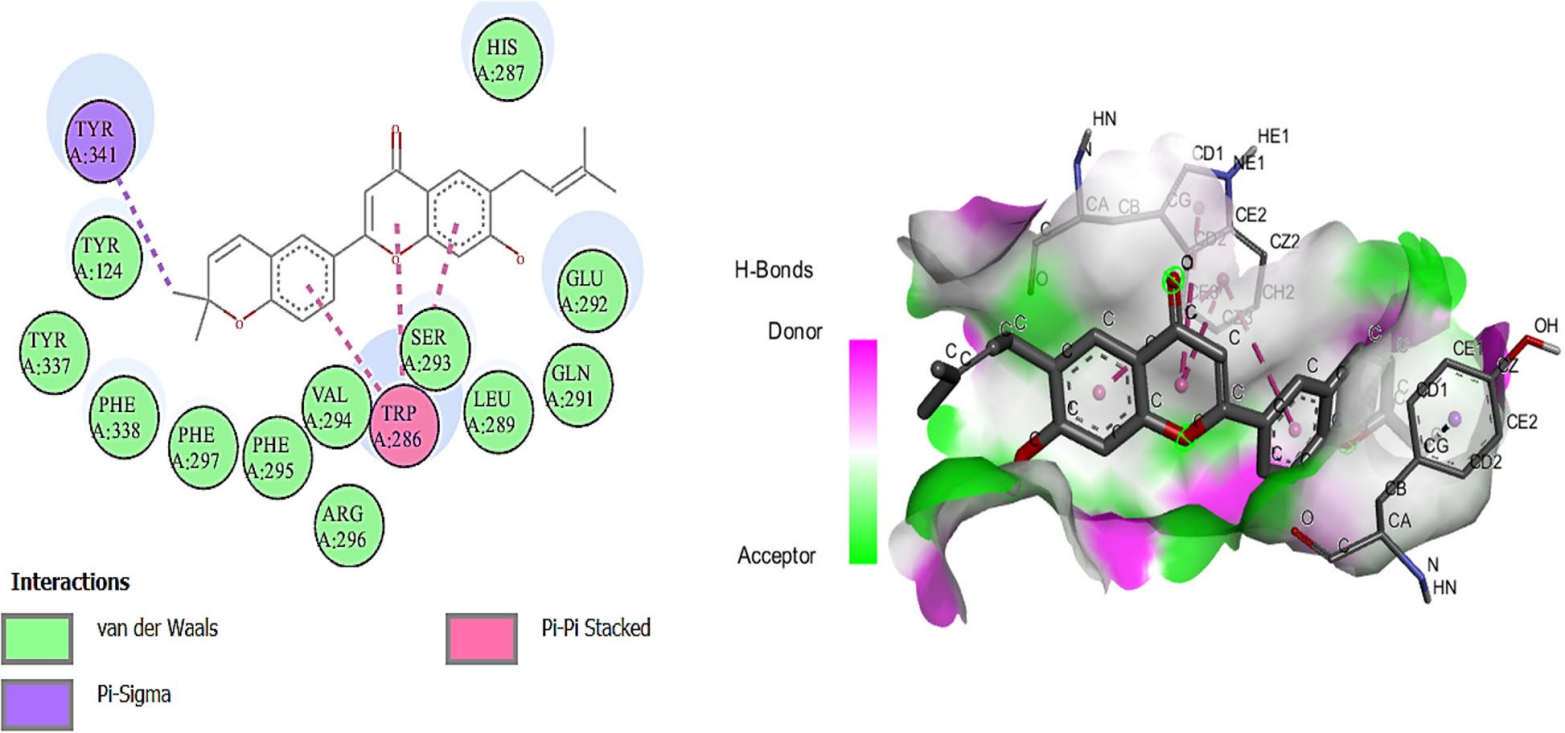
Diagrammatic presentation of 2D (left) and 3D (right) interactions of Kanzonol E with acetylcholinesterase active site residues

Among the docked compounds against acetylcholinesterase enzyme the ligand N-acetyldehydroanonaine and kanzonol E showed the highest binding affinity due to the lowest binding energies (-10.0 kcal/mol) compared to standard inhibitor (galantamine; -7.0 kcal/mol) (Fig. 3). Other ligands showed binding energies in the range -9.3 to -6.1 kcal/mol. While three ligands (quinic acid; -6.1, Rutacultin; -6.5, and Methylsyringin; -6.7 kcal/mol) showed binding affinity weaker than the standard (Table 5).

**Table 5:** Binding energies of identified compounds after docking with enzymes

Sr.No	Compounds	AChE	BChE	ɑ-Amylase	ɑ-glucosidase	Tyrosinase
1	6-Caffeoylsucrose	-7.8	-8.8	-7.3	-8.1	-7.7
2	N-Acetyldehydroanonaine	-10.0	-10.9	-9.4	-8.7	-8.3
3	Bryaflavan	-8.0	-7.7	-8.0	-7.0	-7.2
4	Purpuritenin A	-8.6	-8.8	-7.9	-7.6	-6.7
5	3-O-Feruloylquinic acid	-7.5	-8.5	-7.8	-8.2	-6.9
6	Brosimacutin C	-8.5	-9.3	-8.7	-7.9	-7.5
7	Quinic acid	-6.1	-5.9	-5.8	-5.8	-5.3
8	Murrayazolinine	-9.0	-10.6	-9.0	-9,4	-8.1
9	Rutacultin	-6.5	-8.0	-7.0	-6.8	-6.6
10	Castamollissin	-9.2	-9.5	-8.6	-8.6	-8.1
11	9-Acetoxyfukinanolide	-7.7	-8.6	-7.3	-7.6	-6.2
12	Matteucinol	-9.3	8.1	-7.8	-7.4	-7.2
13	Kaempferol 3-*p*-coumarate	-8.7	-10.4	-8.5	-8.3	-9.0
14	Isocordoin	-8.9	-9.0	-8.4	-7.8	-7.3
15	Methylsyringin	-6.7	-7.0	-7.3	-7.3	-6.3
16	Kanzonol E	-10.0	-9.8	-9.6	-9.1	-8.7
17	Standard	-7.0a	-8.8a	-7.6b	-8.3b	-5.4c

Standards: Galantamine (a), acarbose (b), and kojic acid (c). While AChE and BChE represent the acetylcholinesterase and butyrylcholinesterase respectively

**Fig. 4:**
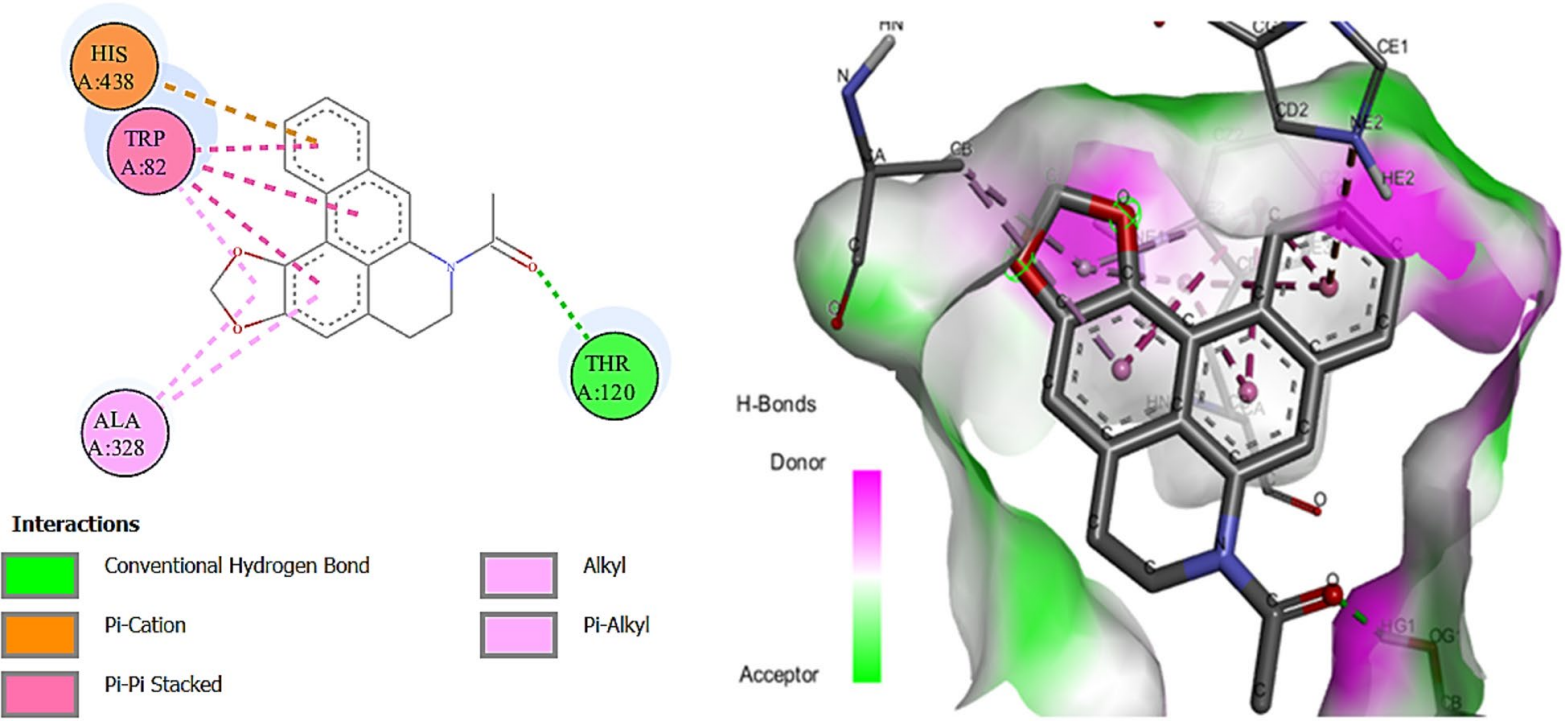
Diagrammatic presentation of 2D (left) and 3D (right) interactions of N-acetyldehydroanonaine E with butyrylcholinesterase active site residues

N-Acetyldehydroanonaine also showed the highest binding affinity among the docked ligands against butyrylcholinesterase enzyme (-10.9 kcal mol) (Fig. 4). Herein, murrayazolinine, kaempferol 3-*p*-coumarate, kanzonol, E castamollissin, brosimacutin C, isocordoin (binding energies; -10.6, -10.4, -9.8, -9.5, -9.3, and -9.3 kcal/mol respectively) exhibited their higher binding affinities towards the enzyme compared to galantamine (-8.8 kcal/mol). While two compounds (6-caffeoylsucrose and purpuritenin A) were showing the binding affinity similar to the standard drug.

**Fig. 5:**
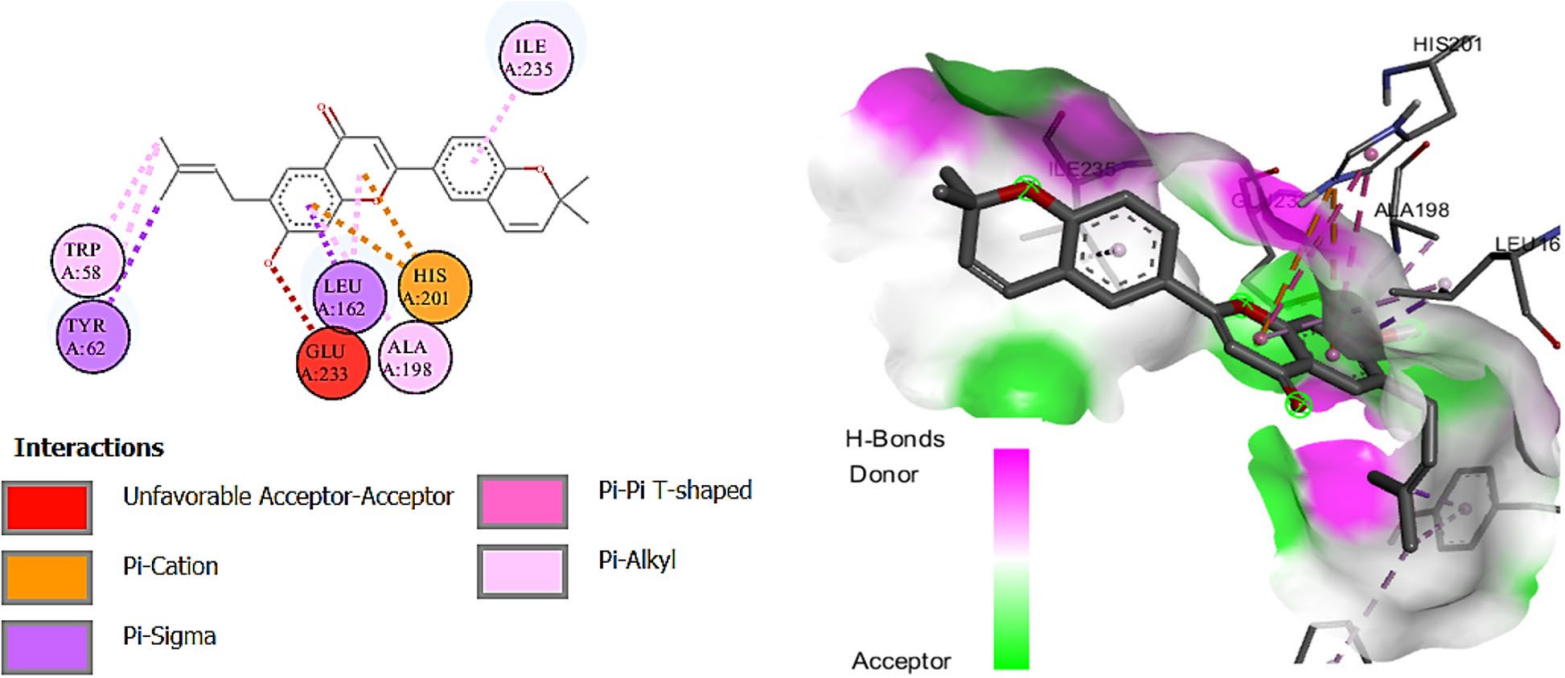
Diagrammatic presentation of 2D (left) and 3D (right) interactions of Kanzonol E with ɑ-amylase active site residues

Eleven of the docked ligands showed ɑ-amylase inhibitory properties due to their lower binding energies compared to acarbose (standard; -7.6 kcal/mol). Kanzonol E showed the highest binding affinity to bind with the enzyme compared to all other docked ligands due to its lowest biding energy (-9.6 kcal/mol) (Fig. 5). While, five ligands expressed more binding energies than standard drug and showed represent their less contribution in the inhibition of the enzyme.

**Fig. 6:**
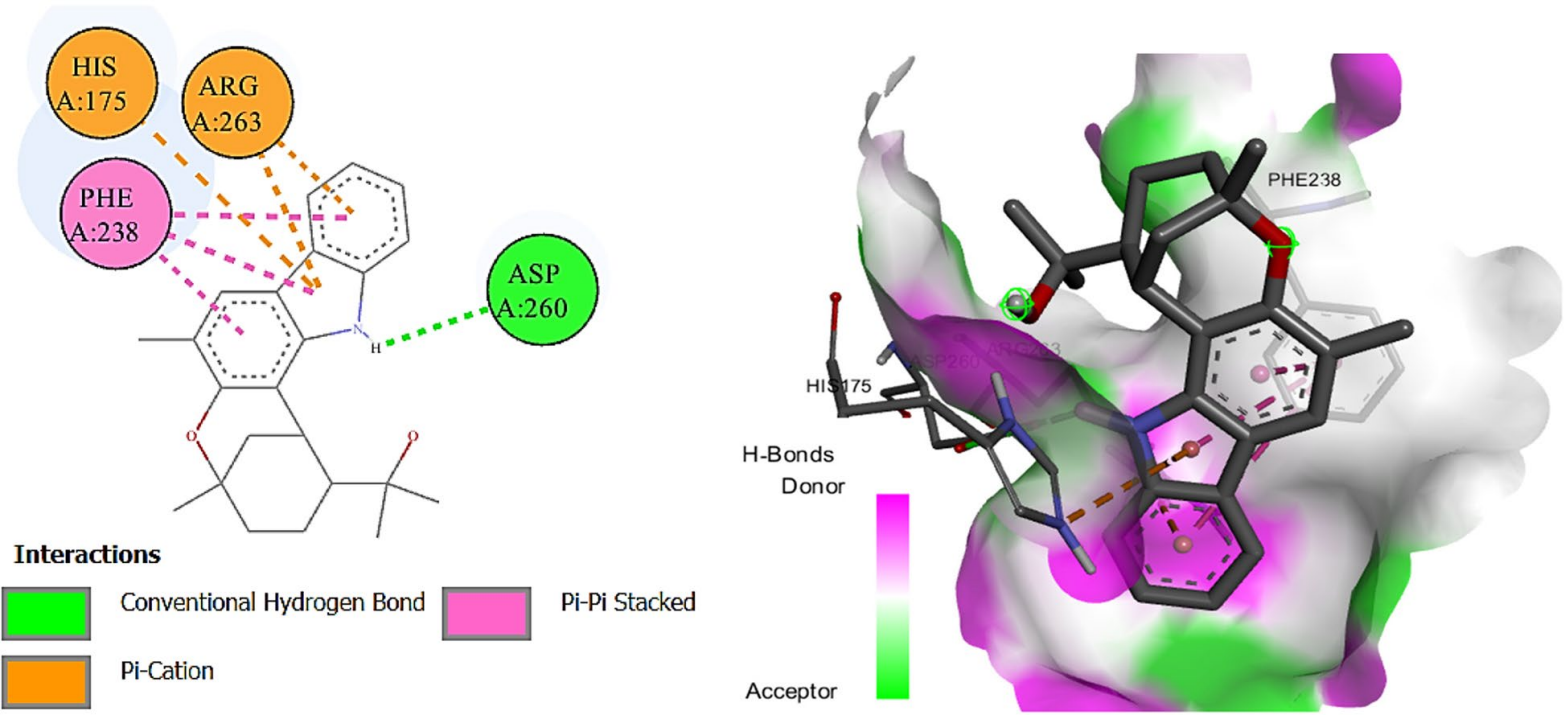
Diagrammatic presentation of 2D (left) and 3D (right) interactions of murrayazolinine with ɑ-glucosidase active site residues

Four compounds exhibited higher binding affinities when docked against the ɑ-glucosidase enzyme, which attributes their contribution to the inhibitory effects of the plant extract. Murrayazolinine showed the highest binding affinity due to -9.4 kcal binding energy compared to the standard inhibitor (acarbose; -8.3 kcal/mol) (Fig. 6), while the compounds kanzonol E (-9.1), N-acetyldehydroanonaine (-8.7), and castamollissin (-8.6) also showed better binding than acarbose. Moreover, kaempferol 3-*p*-coumarate depicted similar binding affinity to the standard and all the remaining compounds showed lesser affinity due to their higher binding energies (-5.8 to -8.2 kcal/mol) than the standard drug.

**Fig. 7:**
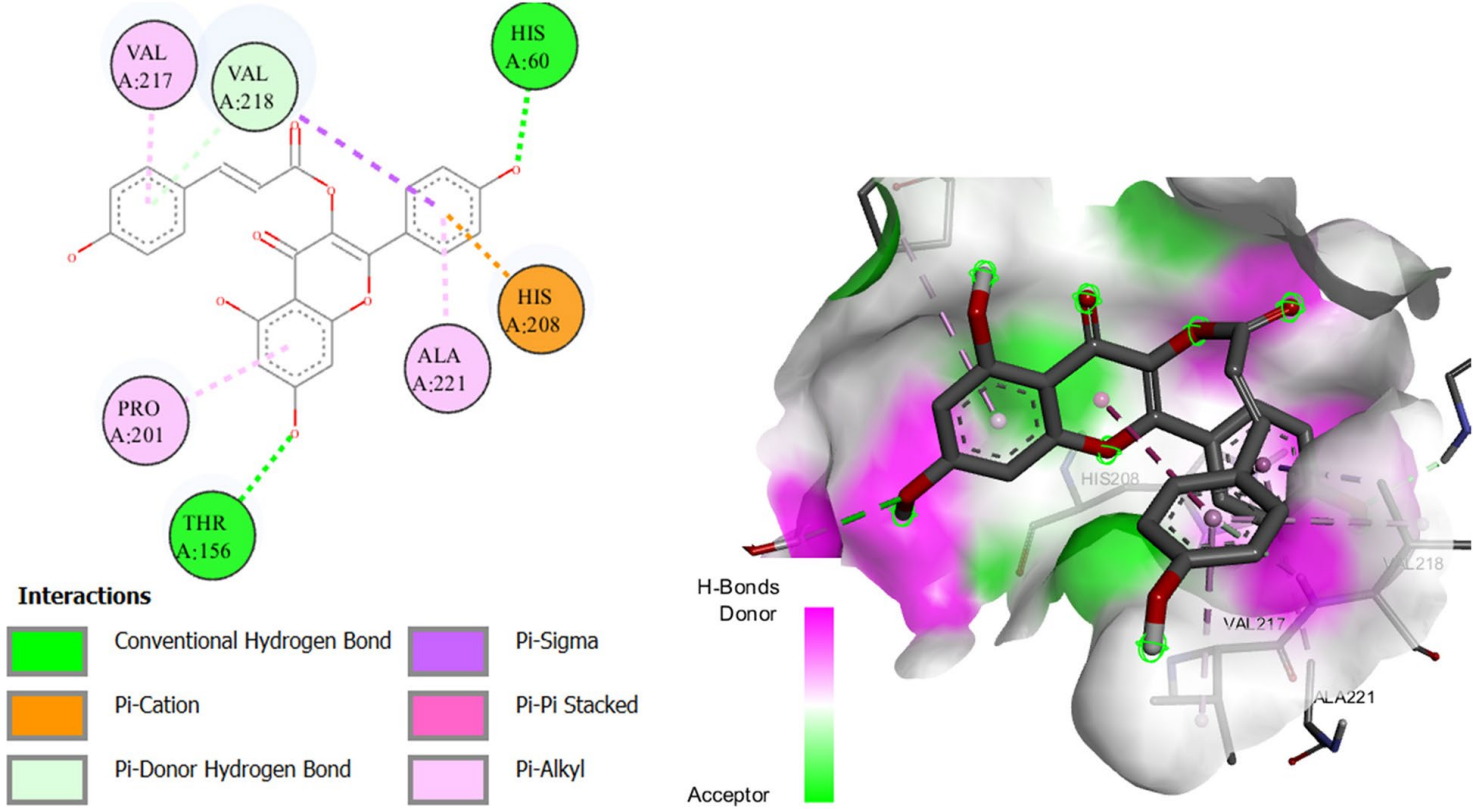
Diagrammatic presentation of 2D (left) and 3D (right) interactions of kaempferol 3-p-coumarate with tyrosinase active site residues

For tyrosinase inhibition only quinic acid (-5.3 kcal/mol) showed lesser binding compared to the kojic acid (-5.4 kcal/mol) used as standard tyrosinase inhibitor. While, all the other docked ligands exhibited lower binding energies (-6.2 to -9.0 kcal/mol) showing their possible contribution in the tyrosinase inhibitory properties of the extract. The results further showed that kaempferol 3-*p*-coumarate has highest binding affinity towards tyrosinase enzyme due to its lowest binding energy (-9.0 kcal/mol) (Fig. 7).

### ADME Analysis

**Fig. 8:**
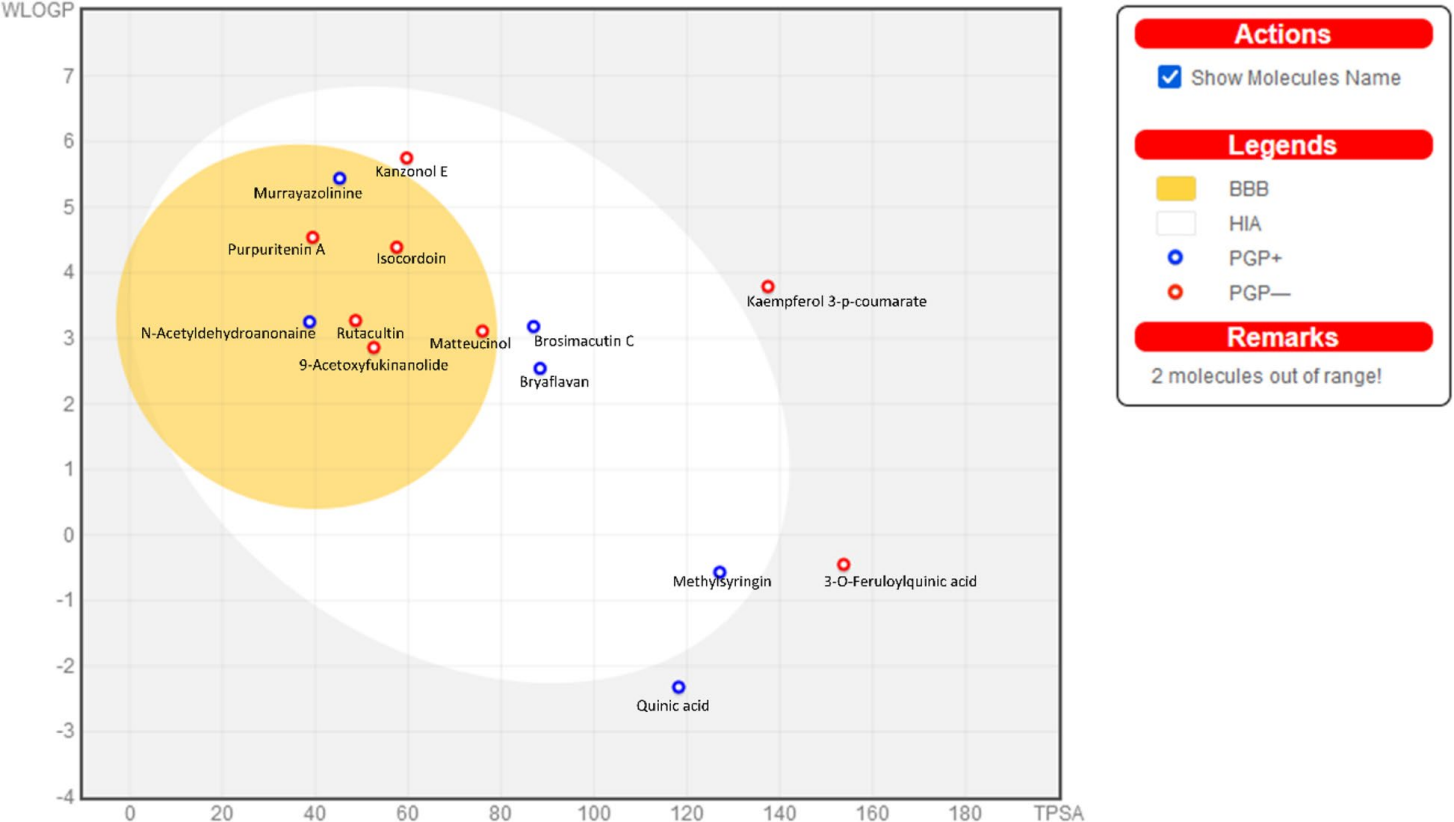
Graphical Distribution of compounds according to predicted model of BOILED-Egg

The information anticipated for the medicinal chemistry, pharmacokinetics, lipophilicity, physicochemical properties, solubility, and drug resemblance of compounds assessed by SwissADME [70] is provided in Table 6. The molecular weights of the docked compounds were found within the range of 200–600 Da and to be 192.17–504.44, based on Lipinski's rule of five. The logP values were between -0.12 to 4.42, which were less than 5. All of the compound's products had an HBA number of 2 to 9 except for two compounds 6-caffeoylsucrose and castamollissin which can accept 14 and 13 HBA respectively. While the same trend was observed for HBD numbers the two compounds have HBD numbers 9 and 8 respectively while the remaining compounds have HBD numbers ≤ 5 [71]. The graphical model known as the Brain Or IntestinaL EstimateD Permeation (BOILED-Egg) technique determines the polarity and lipophilicity of small compounds. Concerning the possibility of oral absorption of medication candidates, this prediction offers a visual cue for the synthesis design of novel compounds [72]. Figure 8 displays a graphic estimate of these selectively docked compounds' gastrointestinal absorption and blood–brain barrier (BBB) penetration. The compounds N-Acetyldehydroanonaine, Purpuritenin A, Murrayazolinine, Rutacultin, 9-Acetoxyfukinanolide and Matteucinol were found in the BBB, while Bryaflavan, Brosimacutin C, Methylsyringin and Kanzonol E were found in white region. The white region contains those compounds which have good potential to be absorbed through the gastrointestinal tract. The compounds 3-O-Feruloylquinic acid, Quinic acid and Kaempferol 3-*p*-coumarate as indicated by the BOILED-Egg plot were presented in gray region. The gray region is designated for poor intestine absorption. The two compounds 6-caffeoylsucrose and castamollissin violated Lipinski's rule and were not shown in BOILED-Egg The compounds showed with blue spot, was discovered to be indicative of their high bioavailability. The compounds Bryaflavan, Brosimacutin C, Methylsyringin and Kanzonol E show great promise as gastrointestinal tract absorbers since they do not cross the blood–brain barrier. These substances have no adverse effects on central nervous system depression or sleepiness because they cannot penetrate the blood–brain barrier.

**Table 6:** Medicinal and Drug-like properties of selected compounds predicted using SwissADME

Compounds	Physicochemical Properties	Lipophilicity	Water Solubility	Pharmacokinetics	Drug likeness	Medicinal Chemistry
6-Caffeoylsucrose	Formula: C_21_H_28_O_14_ Molecular weight: 504.44 g/mol Num. heavy atoms: 35 Num. arom. heavy atoms: 6 Fraction Csp3: 0.57 Num. rotatable bonds: 9 Num. H-bond acceptors: 14 Num. H-bond donors:9 Molar Refractivity: 111.55 TPSA: 236.06	Log Po/w (iLOGP): 1.77 Log Po/w (XLOGP3): -2.30 Log Po/w (WLOGP): -3.83 Log Po/w (MLOGP): -3.59 Log Po/w (SILICOS-IT): -2.65 Consensus Log Po/w: -2.12	Log S (ESOL): -1.05 Solubility: 4.48e + 01 mg/ml; 8.88e-02 mol/l Class: Very soluble Log S (Ali): -2.12 Solubility: 3.81e + 00 mg/ml; 7.56e-03 mol/l Class: Soluble Log S (SILICOS-IT): 2.10 Solubility: 6.39e + 04 mg/ml; 1.27e + 02 mol/l Class: Soluble	GI absorption: Low BBB permeant: No P-gp substrate: No CYP1A2 inhibitor: No CYP2C19 inhibitor: No CYP2C9 inhibitor: No CYP2D6 inhibitor: No CYP3A4 inhibitor: No Log Kp (skin permeation): -11.01 cm/s	Lipinski: No; 3 violations: MW > 500, NorO > 10, NHorOH > 5 Ghose: No; 2 violations: MW > 480, WLOGP < -0.4 Veber: No; 1 violation: TPSA > 140 Egan: No; 1 violation: TPSA > 131.6 Muegge: No; 4 violations: XLOGP3 < -2, TPSA > 150, H-acc > 10, H-don > 5 Bioavailability Score: 0.17	PAINS: 1 alert: catechol_A Brenk: 2 alerts: catechol, michael_acceptor_1 Leadlikeness: No; 2 violations: MW > 350, Rotors > 7 Synthetic accessibility: 5.56
N-Acetyldehydroanonaine	Formula: C_19_H_15_NO_3_ SMolecular weight: 305.33 g/mol Num. heavy atoms: 23 Num. arom. heavy atoms: 14 Fraction Csp3: 0.21 Num. rotatable bonds: 1 Num. H-bond acceptors: 3 Num. H-bond donors: 0 Molar Refractivity: 92.52 TPSA: 38.77	Log Po/w (iLOGP): 2.93 Log Po/w (XLOGP3): 3.63 Log Po/w (WLOGP): 3.25 Log Po/w (MLOGP): 2.89 Log Po/w (SILICOS-IT): 3.88 Consensus Log Po/w: 3.32	Log S (ESOL): -4.40 Solubility: 1.20e-02 mg/ml; 3.94e-05 mol/l Class: Moderately soluble Log S (Ali): -4.13 Solubility: 2.25e-02 mg/ml; 7.38e-05 mol/l Class: Moderately soluble Log S (SILICOS-IT): -5.71 Solubility: 5.94e-04 mg/ml; 1.95e-06 mol/l Class: Moderately soluble	GI absorption: High BBB permeant: Yes P-gp substrate: Yes CYP1A2 inhibitor: Yes CYP2C19 inhibitor: Yes CYP2C9 inhibitor: Yes CYP2D6 inhibitor: No CYP3A4 inhibitor: Yes Log Kp (skin permeation): -5.59 cm/s	Lipinski: Yes; 0 violation Ghose: Yes Veber: Yes Egan: Yes Muegge: Yes Bioavailability Score: 0.55	PAINS: 0 alert Brenk: 1 alert: polycyclic_aromatic_hydrocarbon_3 Leadlikeness: No; 1 violation: XLOGP3 > 3.5 Synthetic accessibility: 2.53
Bryaflavan	Formula: C_17_H_18_O_6_ Molecular weight: 318.32 g/mol Num. heavy atoms: 23 Num. arom. heavy atoms: 12 Fraction Csp3: 0.29 Num. rotatable bonds: 3 Num. H-bond acceptors: 6 Num. H-bond donors:3 Molar Refractivity: 84.13 TPSA: 88.38	Log Po/w (iLOGP): 2.43 Log Po/w (XLOGP3): 2.55 Log Po/w (WLOGP): 2.54 Log Po/w (MLOGP): 1.00 Log Po/w (SILICOS-IT): 2.44 Consensus Log Po/w: 2.19	Log S (ESOL): -3.61 Solubility: 7.85e-02 mg/ml; 2.47e-04 mol/l Class: Soluble Log S (Ali): -4.05 Solubility: 2.82e-02 mg/ml; 8.84e-05 mol/l Class: Moderately soluble Log S (SILICOS-IT): -3.77 Solubility: 5.47e-02 mg/ml; 1.72e-04 mol/l Class: Soluble	GI absorption: High BBB permeant: No P-gp substrate: Yes CYP1A2 inhibitor: Yes CYP2C19 inhibitor: No CYP2C9 inhibitor: No CYP2D6 inhibitor: Yes CYP3A4 inhibitor: Yes Log Kp (skin permeation): -6.43 cm/s	Lipinski: Yes; 0 violation Ghose: Yes Veber: Yes Egan: Yes Muegge: Yes Bioavailability Score: 0.55	PAINS: 1 alert: catechol_A Brenk: 1 alert: catechol Leadlikeness: Yes Synthetic accessibility: 3.41
Purpuritenin A	Formula: C_19_H_16_O_3_ Molecular weight: 292.33 g/mol Num. heavy atoms: 22 Num. arom. heavy atoms: 15 Fraction Csp3: 0.11 Num. rotatable bonds: 4 Num. H-bond acceptors: 3 Num. H-bond donors: 0 Molar Refractivity: 87.48 TPSA: 39.44	Log Po/w (iLOGP): 3.14 Log Po/w (XLOGP3): 4.43 Log Po/w (WLOGP): 4.54 Log Po/w (MLOGP): 2.59 Log Po/w (SILICOS-IT): 4.94 Consensus Log Po/w: 3.93	Log S (ESOL): -4.68 Solubility: 6.05e-03 mg/ml; 2.07e-05 mol/l Class: Moderately soluble Log S (Ali): -4.98 Solubility: 3.09e-03 mg/ml; 1.06e-05 mol/l Class: Moderately soluble Log S (SILICOS-IT): -6.35 Solubility: 1.31e-04 mg/ml; 4.47e-07 mol/l Class: Poorly soluble	GI absorption: High BBB permeant: Yes P-gp substrate: No CYP1A2 inhibitor: Yes CYP2C19 inhibitor: Yes CYP2C9 inhibitor: Yes CYP2D6 inhibitor: No CYP3A4 inhibitor: No Log Kp (skin permeation): -4.94 cm/s	Lipinski: Yes; 0 violation Ghose: Yes Veber: Yes Egan: Yes Muegge: Yes Bioavailability Score: 0.55	PAINS: 0 alert Brenk: 1 alert: michael_acceptor_1 Leadlikeness: No; 1 violation: XLOGP3 > 3.5 Synthetic accessibility: 3.13
3-O-Feruloylquinic acid	Formula: C_17_H_20_O_9_ Molecular weight: 368.34 g/mol Num. heavy atoms: 26 Num. arom. heavy atoms: 6 Fraction Csp3: 0.41 Num. rotatable bonds: 6 Num. H-bond acceptors: 9 Num. H-bond donors: 5 Molar Refractivity: 87.97 TPSA: 153.75	Log Po/w (iLOGP): 1.47 Log Po/w (XLOGP3): -0.10 Log Po/w (WLOGP): -0.45 Log Po/w (MLOGP): -0.81 Log Po/w (SILICOS-IT): -0.07 Consensus Log Po/w: 0.01:	Log S (ESOL): -1.84 Solubility: 5.38e + 00 mg/ml; 1.46e-02 mol/l Class: Very soluble Log S (Ali): -2.68 Solubility: 7.76e-01 mg/ml; 2.11e-03 mol/l Class: Soluble Log S (SILICOS-IT): -0.29 Solubility: 1.89e + 02 mg/ml; 5.13e-01 mol/l Class: Soluble	GI absorption: Low BBB permeant: No P-gp substrate: No CYP1A2 inhibitor: No CYP2C19 inhibitor: No CYP2C9 inhibitor: No CYP2D6 inhibitor: No CYP3A4 inhibitor: No Log Kp (skin permeation): -8.62 cm/s	Lipinski: Yes; 0 violation Ghose: No; 1 violation: WLOGP < -0.4 Veber: No; 1 violation: TPSA > 140 Egan: No; 1 violation: TPSA > 131.6 Muegge: No; 1 violation: TPSA > 150 Bioavailability Score: 0.11	PAINS: 0 alert Brenk: 1 alert: michael_acceptor_1 Leadlikeness: No; 1 violation: MW > 350 Synthetic accessibility: 4.25
Brosimacutin C	Formula: C_20_H_22_O_5_ Molecular weight: 342.39 g/mol Num. heavy atoms: 25 Num. arom. heavy atoms: 12 Fraction Csp3: 0.35 Num. rotatable bonds: 4 Num. H-bond acceptors: 5 Num. H-bond donors: 3 Molar Refractivity: 94.94 TPSA: 86.99	Log Po/w (iLOGP): 2.62 Log Po/w (XLOGP3): 2.75 Log Po/w (WLOGP): 3.18 Log Po/w (MLOGP): 1.63 Log Po/w (SILICOS-IT): 3.62 Consensus Log Po/w: 2.76	Log S (ESOL): -3.79 Solubility: 5.60e-02 mg/ml; 1.63e-04 mol/l Class: Soluble Log S (Ali): -4.23 Solubility: 2.01e-02 mg/ml; 5.87e-05 mol/l Class: Moderately soluble Log S (SILICOS-IT): -5.02 Solubility: 3.30e-03 mg/ml; 9.64e-06 mol/l Class: Moderately soluble	GI absorption: High BBB permeant: No P-gp substrate: Yes CYP1A2 inhibitor: No CYP2C19 inhibitor: No CYP2C9 inhibitor: No CYP2D6 inhibitor: Yes CYP3A4 inhibitor: No Log Kp (skin permeation): -6.44 cm/s	Lipinski: Yes; 0 violation Ghose: Yes Veber: Yes Egan: Yes Muegge: Yes Bioavailability Score: 0.55	PAINS: 0 alert Brenk: 0 alert Leadlikeness: Yes Synthetic accessibility: 3.61
Quinic acid	Formula: C_7_H_12_O_6_ Molecular weight: 192.17 g/mol Num. heavy atoms: 13 Num. arom. heavy atoms: 0 Fraction Csp3: 0.86 Num. rotatable bonds: 1 Num. H-bond acceptors: 6 Num. H-bond donors: 5 Molar Refractivity: 40.11 TPSA: 118.22	Log Po/w (iLOGP): -0.12 Log Po/w (XLOGP3): -2.37 Log Po/w (WLOGP): -2.32 Log Po/w (MLOGP): -2.14 Log Po/w (SILICOS-IT): -1.82 Consensus Log Po/w: -1.75	Log S (ESOL): 0.53 Solubility: 6.48e + 02 mg/ml; 3.37e + 00 mol/l Class: Highly soluble Log S (Ali): 0.43 Solubility: 5.12e + 02 mg/ml; 2.66e + 00 mol/l Class: Highly soluble Log S (SILICOS-IT): 2.08 Solubility: 2.30e + 04 mg/ml; 1.20e + 02 mol/l Class: Soluble	GI absorption: Low BBB permeant: No P-gp substrate: Yes CYP1A2 inhibitor: No CYP2C19 inhibitor: No CYP2C9 inhibitor: No CYP2D6 inhibitor: No CYP3A4 inhibitor: No Log Kp (skin permeation): -9.15 cm/s	Lipinski: Yes; 0 violation Ghose: No; 1 violation: WLOGP < -0.4 Veber: Yes Egan: Yes Muegge: No; 2 violations: MW < 200, XLOGP3 < -2 Bioavailability Score: 0.56	PAINS: 0 alert Brenk: 0 alert Leadlikeness: No; 1 violation: MW < 250 Synthetic accessibility: 3.34
Murrayazolinine	Formula: C_23_H_27_NO_2_ Molecular weight: 349.47 g/mol Num. heavy atoms: 26 Num. arom. heavy atoms: 13 Fraction Csp3: 0.48 Num. rotatable bonds: 1 Num. H-bond acceptors: 2 Num. H-bond donors: 2 Molar Refractivity: 107.69 TPSA: 45.25	Log Po/w (iLOGP): 3.42 Log Po/w (XLOGP3): 4.84 Log Po/w (WLOGP): 5.44 Log Po/w (MLOGP): 3.93 Log Po/w (SILICOS-IT): 5.46 Consensus Log Po/w: 4.62	Log S (ESOL): -5.36 Solubility: 1.53e-03 mg/ml; 4.37e-06 mol/l Class: Moderately soluble Log S (Ali): -5.52 Solubility: 1.05e-03 mg/ml; 2.99e-06 mol/l Class: Moderately soluble Log S (SILICOS-IT): -6.87 Solubility: 4.71e-05 mg/ml; 1.35e-07 mol/l Class: Poorly soluble	GI absorption: High BBB permeant: Yes P-gp substrate: Yes CYP1A2 inhibitor: No CYP2C19 inhibitor: No CYP2C9 inhibitor: No CYP2D6 inhibitor: Yes CYP3A4 inhibitor: No Log Kp (skin permeation): -5.00 cm/s	Lipinski: Yes; 0 violation Ghose: Yes Veber: Yes Egan: Yes Muegge: Yes Bioavailability Score: 0.55	PAINS: 0 alert Brenk: 0 alert Leadlikeness: No; 1 violation: XLOGP3 > 3.5 Synthetic accessibility: 4.53
Rutacultin	Formula: C_16_H_18_O_4_ Molecular weight: 274.31 g/mol Num. heavy atoms: 20 Num. arom. heavy atoms: 10 Fraction Csp3: 0.31 Num. rotatable bonds: 4 Num. H-bond acceptors: 4 Num. H-bond donors: 0 Molar Refractivity: 79.07 TPSA: 48.67	Log Po/w (iLOGP): 3.25 Log Po/w (XLOGP3): 3.81 Log Po/w (WLOGP): 3.27 Log Po/w (MLOGP): 2.29 Log Po/w (SILICOS-IT): 4.09 Consensus Log Po/w: 3.34	Log S (ESOL): -4.05 Solubility: 2.46e-02 mg/ml; 8.97e-05 mol/l Class: Moderately soluble Log S (Ali): -4.53 Solubility: 8.15e-03 mg/ml; 2.97e-05 mol/l Class: Moderately soluble Log S (SILICOS-IT): -5.13 Solubility: 2.01e-03 mg/ml; 7.33e-06 mol/l Class: Moderately soluble	GI absorption: High BBB permeant: Yes P-gp substrate: No CYP1A2 inhibitor: Yes CYP2C19 inhibitor: Yes CYP2C9 inhibitor: Yes CYP2D6 inhibitor: No CYP3A4 inhibitor: No Log Kp (skin permeation): -5.27 cm/s	Lipinski: Yes; 0 violation Ghose: Yes Veber: Yes Egan: Yes Muegge: Yes Bioavailability Score: 0.55	PAINS: 0 alert Brenk: 2 alerts: cumarine, isolated_alkene Leadlikeness: No; 1 violation: XLOGP3 > 3.5 Synthetic accessibility: 3.27
Castamollissin	Formula: C_20_H_20_O_13_ Molecular weight: 468.37 g/mol Num. heavy atoms: 33 Num. arom. heavy atoms: 12 Fraction Csp3: 0.30 Num. rotatable bonds: 7 Num. H-bond acceptors: 13 Num. H-bond donors: 8 Molar Refractivity: 105.73 TPSA: 223.67 Å^2^	Log Po/w (iLOGP): 0.93 Log Po/w (XLOGP3): -0.87 Log Po/w (WLOGP): -0.93 Log Po/w (MLOGP): -2.37 Log Po/w (SILICOS-IT): -1.34 Consensus Log Po/w: -0.92	Log S (ESOL): -2.00 Solubility: 4.65e + 00 mg/ml; 9.93e-03 mol/l Class: Soluble Log S (Ali): -3.35 Solubility: 2.11e-01 mg/ml; 4.51e-04 mol/l Class: Soluble Log S (SILICOS-IT): 0.07 Solubility: 5.49e + 02 mg/ml; 1.17e + 00 mol/l Class: Soluble	GI absorption: Low BBB permeant: No P-gp substrate: No CYP1A2 inhibitor: No CYP2C19 inhibitor: No CYP2C9 inhibitor: No CYP2D6 inhibitor: No CYP3A4 inhibitor: No Log Kp (skin permeation): -9.77 cm/s	Lipinski: No; 2 violations: NorO > 10, NHorOH > 5 Ghose: No; 1 violation: WLOGP < -0.4 Veber: No; 1 violation: TPSA > 140 Egan: No; 1 violation: TPSA > 131.6 Muegge: No; 3 violations: TPSA > 150, H-acc > 10, H-don > 5 Bioavailability Score: 0.17	PAINS: 1 alert: catechol_A Brenk: 2 alerts: aldehyde, catechol Leadlikeness: No; 1 violation: MW > 350 Synthetic accessibility: 4.88
9-Acetoxyfukinanolide	Formula: C_17_H_24_O_4_ Molecular weight: 292.37 g/mol Num. heavy atoms: 21 Num. arom. heavy atoms: 0 Fraction Csp3: 0.76 Num. rotatable bonds: 2 Num. H-bond acceptors: 4 Num. H-bond donors: 0 Molar Refractivity: 79.07 TPSA: 52.60 Å^2^	Log Po/w (iLOGP): 2.48 Log Po/w (XLOGP3): 2.92 Log Po/w (WLOGP): 2.86 Log Po/w (MLOGP): 2.92 Log Po/w (SILICOS-IT): 3.18 Consensus Log Po/w: 2.87	Log S (ESOL): -3.36 Solubility: 1.28e-01 mg/ml; 4.36e-04 mol/l Class: Soluble Log S (Ali): -3.69 Solubility: 6.03e-02 mg/ml; 2.06e-04 mol/l Class: Soluble Log S (SILICOS-IT): -3.35 Solubility: 1.30e-01 mg/ml; 4.45e-04 mol/l Class: Soluble	GI absorption: High BBB permeant: Yes P-gp substrate: No CYP1A2 inhibitor: No CYP2C19 inhibitor: No CYP2C9 inhibitor: No CYP2D6 inhibitor: No CYP3A4 inhibitor: No Log Kp (skin permeation): -6.01 cm/s	Lipinski: Yes; 0 violation Ghose: Yes Veber: Yes Egan: Yes Muegge: Yes Bioavailability Score: 0.55	PAINS: 0 alert Brenk: 2 alerts: isolated_alkene, more_than_2_esters Leadlikeness: Yes Synthetic accessibility: 4.88
Matteucinol	Formula: C_18_H_18_O_5_ Molecular weight: 314.33 g/mol Num. heavy atoms: 23 Num. arom. heavy atoms: 12 Fraction Csp3: 0.28 Num. rotatable bonds: 2 Num. H-bond acceptors: 5 Num. H-bond donors: 2 Molar Refractivity: 85.97 TPSA: 75.99 Å^2^	Log Po/w (iLOGP): 2.95 Log Po/w (XLOGP3): 3.45 Log Po/w (WLOGP): 3.11 Log Po/w (MLOGP): 1.44 Log Po/w (SILICOS-IT): 3.58 Consensus Log Po/w: 2.91	Log S (ESOL): -4.22 Solubility: 1.91e-02 mg/ml; 6.08e-05 mol/l Class: Moderately soluble Log S (Ali): -4.73 Solubility: 5.89e-03 mg/ml; 1.87e-05 mol/l Class: Moderately soluble Log S (SILICOS-IT): -4.88 Solubility: 4.16e-03 mg/ml; 1.32e-05 mol/l Class: Moderately soluble	GI absorption: High BBB permeant: Yes P-gp substrate: No CYP1A2 inhibitor: Yes CYP2C19 inhibitor: Yes CYP2C9 inhibitor: Yes CYP2D6 inhibitor: Yes CYP3A4 inhibitor: Yes Log Kp (skin permeation): -5.77 cm/s	Lipinski: Yes; 0 violation Ghose: Yes Veber: Yes Egan: Yes Muegge: Yes Bioavailability Score: 0.55	PAINS: 0 alert Brenk: 0 alert Leadlikeness: Yes Synthetic accessibility: 3.37
Kaempferol 3-*p*-coumarate	Formula: C_24_H_16_O_8_ Molecular weight: 432.38 g/mol Num. heavy atoms: 32 Num. arom. heavy atoms: 22 Fraction Csp3: 0.00 Num. rotatable bonds: 5 Num. H-bond acceptors: 8 Num. H-bond donors: 4 Molar Refractivity: 117.12 TPSA: 137.43 Å^2^	Log Po/w (iLOGP): 2.67 Log Po/w (XLOGP3): 4.36 Log Po/w (WLOGP): 3.79 Log Po/w (MLOGP): 1.17 Log Po/w (SILICOS-IT): 3.65 Consensus Log Po/w: 3.13	Log S (ESOL): -5.45 Solubility: 1.55e-03 mg/ml; 3.58e-06 mol/l Class: Moderately soluble Log S (Ali): -6.96 Solubility: 4.72e-05 mg/ml; 1.09e-07 mol/l Class: Poorly soluble Log S (SILICOS-IT): -6.02 Solubility: 4.16e-04 mg/ml; 9.62e-07 mol/l Class: Poorly soluble	GI absorption: Low BBB permeant: No P-gp substrate: No CYP1A2 inhibitor: No CYP2C19 inhibitor: No CYP2C9 inhibitor: Yes CYP2D6 inhibitor: No CYP3A4 inhibitor: No Log Kp (skin permeation): -5.84 cm/s	Lipinski: Yes; 0 violation Ghose: Yes Veber: Yes Egan: No; 1 violation: TPSA > 131.6 Muegge: Yes Bioavailability Score: 0.55	PAINS: 0 alert Brenk: 1 alert: michael_acceptor_1 Leadlikeness: No; 2 violations: MW > 350, XLOGP3 > 3.5 Synthetic accessibility: 3.79
Isocordoin	Formula: C_20_H_20_O_3_ Molecular weight: 308.37 g/mol Num. heavy atoms: 23 Num. arom. heavy atoms: 12 Fraction Csp3: 0.15 Num. rotatable bonds: 5 Num. H-bond acceptors: 3 Num. H-bond donors: 2 Molar Refractivity: 94.01 TPSA: 57.53 Å^22^	Log Po/w (iLOGP): 3.39 Log Po/w (XLOGP3): 5.46 Log Po/w (WLOGP): 4.39 Log Po/w (MLOGP): 3.28 Log Po/w (SILICOS-IT): 4.70 Consensus Log Po/w: 4.24	Log S (ESOL): -5.25 Solubility: 1.74e-03 mg/ml; 5.65e-06 mol/l Class: Moderately soluble Log S (Ali): -6.43 Solubility: 1.16e-04 mg/ml; 3.76e-07 mol/l Class: Poorly soluble Log S (SILICOS-IT): -5.06 Solubility: 2.70e-03 mg/ml; 8.77e-06 mol/l Class: Moderately soluble	GI absorption: High BBB permeant: Yes P-gp substrate: No CYP1A2 inhibitor: Yes CYP2C19 inhibitor: Yes CYP2C9 inhibitor: Yes CYP2D6 inhibitor: No CYP3A4 inhibitor: Yes Log Kp (skin permeation): -4.30 cm/s	Lipinski: Yes; 0 violation Ghose: Yes Veber: Yes Egan: Yes Muegge: No; 1 violation: XLOGP3 > 5 Bioavailability Score: 0.55	PAINS: 0 alert Brenk: 2 alerts: isolated_alkene, michael_acceptor_1 Leadlikeness: No; 1 violation: XLOGP3 > 3.5 Synthetic accessibility: 2.99
Methylsyringin	Formula: C_18_H_26_O_9_ Molecular weight: 386.39 g/mol Num. heavy atoms: 27 Num. arom. heavy atoms: 6 Fraction Csp3: 0.56 Num. rotatable bonds: 8 Num. H-bond acceptors: 9 Num. H-bond donors: 4 Molar Refractivity: 94.36 TPSA: 127.07 Å^2^	Log Po/w (iLOGP): 2.46 Log Po/w (XLOGP3): -0.77 Log Po/w (WLOGP): -0.57 Log Po/w (MLOGP): -1.35 Log Po/w (SILICOS-IT): 0.42 Consensus Log Po/w: 0.04	Log S (ESOL): -1.39 Solubility: 1.59e + 01 mg/ml; 4.10e-02 mol/l Class: Very soluble Log S (Ali): -1.42 Solubility: 1.47e + 01 mg/ml; 3.80e-02 mol/l Class: Very soluble Log S (SILICOS-IT): -1.03 Solubility: 3.61e + 01 mg/ml; 9.33e-02 mol/l Class: Soluble	GI absorption: High BBB permeant: No P-gp substrate: Yes CYP1A2 inhibitor: No CYP2C19 inhibitor: No CYP2C9 inhibitor: No CYP2D6 inhibitor: No CYP3A4 inhibitor: No Log Kp (skin permeation): -9.20 cm/s	Lipinski: Yes; 0 violation Ghose: No; 1 violation: WLOGP < -0.4 Veber: Yes Egan: Yes Muegge: Yes Bioavailability Score: 0.55	PAINS: 0 alert Brenk: 0 alert Leadlikeness: No; 2 violations: MW > 350, Rotors > 7 Synthetic accessibility: 4.88
Kanzonol E	Formula: C_25_H_24_O_4_ Molecular weight: 388.46 g/mol Num. heavy atoms: 29 Num. arom. heavy atoms: 16 Fraction Csp3: 0.24 Num. rotatable bonds: 3 Num. H-bond acceptors: 4 Num. H-bond donors: 1 Molar Refractivity: 117.78 TPSA: 59.67 Å^2^	Log Po/w (iLOGP): 4.24 Log Po/w (XLOGP3): 5.67 Log Po/w (WLOGP): 5.75 Log Po/w (MLOGP): 3.20 Log Po/w (SILICOS-IT): 6.14 Consensus Log Po/w: 5.00	Log S (ESOL): -6.03 Solubility: 3.62e-04 mg/ml; 9.32e-07 mol/l Class: Poorly soluble Log S (Ali): -6.69 Solubility: 7.97e-05 mg/ml; 2.05e-07 mol/l Class: Poorly soluble Log S (SILICOS-IT): -7.62 Solubility: 9.34e-06 mg/ml; 2.40e-08 mol/l Class: Poorly soluble	GI absorption: High BBB permeant: No P-gp substrate: No CYP1A2 inhibitor: No CYP2C19 inhibitor: Yes CYP2C9 inhibitor: Yes CYP2D6 inhibitor: No CYP3A4 inhibitor: No Log Kp (skin permeation): -4.64 cm/s	Lipinski: Yes; 0 violation Ghose: No; 1 violation: WLOGP > 5.6 Veber: Yes Egan: Yes Muegge: No; 1 violation: XLOGP3 > 5 Bioavailability Score: 0.55	PAINS: 0 alert Brenk: 1 alert: isolated_alkene Leadlikeness: No; 2 violations: MW > 350, XLOGP3 > 3.5 Synthetic accessibility: 4.14

## Data Availability

The datasets used during the current study available from the corresponding author on reasonable request.

## References

[1] Tsevegsuren N, Fujimoto K, Christie WW, Endo Y (2003). Occurrence of a novel cis, cis, cis-octadeca-3,9,12-trienoic (Z,Z,Z-octadeca-3,9,12-trienoic) acid in Chrysanthemum (tanacetum) zawadskii herb seed oil. Lipids.

[2] Bano A, Ahmad M, Hadda TB, Saboor A, Sultana S, Zafar M, Khan MPZ, Arshad M, Ashraf MA (2014). Quantitative ethnomedicinal study of plants used in the skardu valley at high altitude of Karakoram-Himalayan range, Pakistan Pakistan. J Ethnobiol Ethnomed.

[3] Khan SW, Abbas Q, Hassan SN, Khan H, Hussain A (2015). Medicinal Plants of Turmic Valley (Central Karakoram National Park), Gilgit-Baltistan, Pakistan. J Biores Manage.

[4] Chawla A, Rajkumar S, Singh K, Lal B, Singh R, Thukral A (2008). Plant species diversity along an altitudinal gradient of Bhabha Valley in western Himalaya. J Mt Sci.

[5] Abbas Z, Khan SM, Alam J, Khan SW, Abbasi AM (2017). Medicinal plants used by inhabitants of the Shigar Valley Baltistan region of Karakorum range-Pakistan. J Ethnobiol Ethnomed.

[6] Molina-Torres J, Garcia-Chavez A, Ramirez-Chavez E (1999). Antimicrobial properties of alkamides present in flavouring plants traditionally used in Mesoamerica: affinin and capsaicin. J Ethnopharmacol.

[7] Shahhoseini R, Azizi M, Asili J, Moshtaghi N, Samiei L (2019). Comprehensive assessment of phytochemical potential of Tanacetum parthenium (L.): phenolic compounds, antioxidant activity, essential oil and parthenolide. J Essential Oil Bearing Plants.

[8] Ali Ş, Kürkçüoğlu M, Bitiş L, Doğan A, Başer K (2020). Essential oil composition of different parts of Tanacetum cilicicum (Boiss.) Grierson. Nat Volatiles Essential Oils.

[9] Abad M, Bermejo P, Villar A (1995). An approach to the genus Tanacetum L. (Compositae): phytochemical and pharmacological review. Phytother Res.

[10] Holland R, Hendriks JH, Vebeek AL, Mravunac M, Schuurmans Stekhoven JH (1990). Extent, distribution, and mammographic/histological correlations of breast ductal carcinoma in situ. Lancet.

[11] Hitmi A, Coudret A, Barthomeuf C (2000). The production of pyrethrins by plant cell and tissue cultures of Chrysanthemum cinerariaefolium and Tagetes species. Crit Rev Biochem Mol Biol.

[12] Sadique J, Chandra T, Thenmozhi V, Elango V (1987). The anti-inflammatory activity of Enicostemma littorale and Mollugo cerviana. Biochem Med Metab Biol.

[13] Mladenova K, Tsankova E, van Hung D (1988). New sesquiterpenoids from Chrysanthemum indicum var. tuneful. Planta Med.

[14] Kumar V, Tyagi D (2013). Chemical composition and biological activities of essential oils of genus Tanacetum-a review. J Pharmacognosy Phytochem.

[15] Chavez ML, Chavez PI (1999). Feverfew. Hosp Pharm.

[16] Chavez F, Strutton P, Friederich G, Feely R, Feldman G, Foley D, McPhaden M (1999). Biological and chemical response of the equatorial Pacific Ocean to the 1997–98 El Niño. Science.

[17] Hwang SH, Kim HY, Quispe YNG, Wang Z, Zuo G, Lim SS (2019). Aldose Reductase, Protein Glycation inhibitory and antioxidant of Peruvian medicinal plants: the case of Tanacetum parthenium L. and its constituents. Molecules.

[18] Schinella GR, Giner RM, Recio MC, Mordujovich de Buschiazzo P, Rios JL, Manez S (1998). Anti-inflammatory effects of South American Tanacetum vulgare. J Pharm Pharmacol.

[19] Mordujovich-Buschiazzo P, Balsa E, Buschiazzo H, Mandrile E, Rosella M (1996). Anti-inflammatory activity of Tanacetum vulgare. Fitoterapia.

[20] Bagci E, Kursat M, Kocak A, Gur S (2008). Composition and antimicrobial activity of the essential oils of Tanacetum balsamita L. subsp balsamita and T. chiliophyllum (Fisch. et Mey.) Schultz Bip var chiliophyllum (Asteraceae) from Turkey. J Essential Oil Bearing Plants.

[21] Keskitalo M, Pehu E, Simon JE (2001). Variation in volatile compounds from tansy (Tanacetum vulgare L.) related to genetic and morphological differences of genotypes. Biochem Syst Ecol.

[22] Bandonien D, Pukalskas A, Venskutonis P, Gruzdien D (2000). Preliminary screening of antioxidant activity of some plant extracts in rapeseed oil. Food Res Int.

[23] Petrovic SD, Dobric S, Bokonjic D, Niketic M, Garcia-Pineres A, Merfort I (2003). Evaluation of Tanacetum larvatum for an anti-inflammatory activity and for the protection against indomethacin-induced ulcerogenesis in rats. J Ethnopharmacol.

[24] Tournier H, Schinella G, de Balsa EM, Buschiazzo H, Manez S, Mordujovich de Buschiazzo P (1999). Effect of the chloroform extract of Tanacetum vulgare and one of its active principles, parthenolide, on experimental gastric ulcer in rats. J Pharm Pharmacol.

[25] Kuusik A, Tartes U, Harak M, Hiiesaar K, Metspalu L (2013). Developmental changes during metamorphosis in Tenebrio molitor (Coleoptera: Tenebrionidae) studied by calorimetric thermography. EJE.

[26] Vukic MD, Vukovic NL, Obradovic AD, Galovičová L, Čmiková N, Kačániová M, Matic MM (2022). Chemical composition and biological activity of Tanacetum balsamita essential oils obtained from different plant organs. Plants.

[27] Tiuman TS, Ueda-Nakamura T, Garcia Cortez DA, Dias Filho BP, Morgado-Diaz JA, de Souza W, Nakamura CV (2005). Antileishmanial activity of parthenolide, a sesquiterpene lactone isolated from Tanacetum parthenium. Antimicrob Agents Chemother.

[28] Pillay P, Maharaj VJ, Smith PJ (2008). Investigating South African plants as a source of new antimalarial drugs. J Ethnopharmacol.

[29] Ismail M, Kowsar A, Javed S, Choudhary MI, Khan SW, Abbas Q, Tang Y, Wang W (2021). The Antibacterial, Insecticidal and Nematocidal Activities and Toxicity Studies of Tanacetum falconeri Hook. F. Turk J Pharm Sci.

[30] Shazmeen N, Nazir M, Riaz N, Saleem M, Tousif MI, Tauseef S, Uddin R, Mukhtar M, Zengin G, Mollica A (2022). In vitro antioxidant and enzyme inhibitory studies, computational analysis and chemodiversity of an emergency food plant Caralluma edulis (Edgew.) Benth. ex Hook. f: A multifunctional approach to provide new ingredients for nutraceuticals and functional foods. Food Bioscience.

[31] Khan J, Tousif MI, Saleem M, Nazir M, Touseef S, Saleem K, Asim S, Khan A, Asghar MA, Zengin G (2021). Insight into the phytochemical composition, biological activities and docking studies of Moringa oleifera Lam. to authenticate its use in biopharmaceutical industries. Industr Crops Prod.

[32] Saleem M, Shazmeen N, Nazir M, Riaz N, Zengin G, Ataullah HM, Qurat Ul A, Nisar F, Mukhtar M, Tousif MI (2021). Investigation on the phytochemical composition, antioxidant and enzyme inhibition potential of Polygonum Plebeium R. Br: a comprehensive approach to disclose new nutraceutical and functional food ingredients. Chem Biodivers.

[33] Tousif MI, Nazir M, Saleem M, Tauseef S, Uddin R, Altaf M, Zengin G, Ak G, Ozturk RB, Mahomoodally MF (2022). Exploring the industrial importance of a miracle herb Withania somnifera (L.) Dunal: Authentication through chemical profiling, in vitro studies and computational analyses. Process Biochem.

[34] Ahmed M, Ahmad S, Aati HY, Sherif AE, Ashkan MF, Alrahimi J, Motwali EA, Tousif MI, Khan MA, Hussain M (2022). Phytochemical, antioxidant, enzyme inhibitory, thrombolytic, antibacterial, antiviral and in silico studies of Acacia jacquemontii leaves. Arab J Chem.

[35] Ahmed M, Khan K-U-R, Ahmad S, Aati HY, Ovatlarnporn C, Rehman MS-U, Javed T, Khursheed A, Ghalloo BA, Dilshad R (2022). Comprehensive phytochemical profiling, biological activities, and molecular docking studies of Pleurospermum candollei: An insight into potential for natural products development. Molecules.

[36] Tepe B, Sokmen A (2007). Screening of the antioxidative properties and total phenolic contents of three endemic Tanacetum subspecies from Turkish flora. Bioresour Technol.

[37] Emre I (2021). The biochemical content and antioxidant capacities of endemic Tanacetum densum (Lab.) Schultz Bip. Subsp. laxum, and Tanacetum densum (Lab.) Schultz Bip. Subsp. amani Heywood growing in Turkey. Braz J Biol.

[38] Babich O, Larina V, Krol O, Ulrikh E, Sukhikh S, Gureev MA, Prosekov A, Ivanova S (2023). In vitro study of biological activity of Tanacetum vulgare Extracts. Pharmaceutics.

[39] Gevrenova R, Zengin G, Sinan KI, Zheleva-Dimitrova D, Balabanova V, Kolmayer M, Voynikov Y, Joubert O (2022). An in-depth study of metabolite profile and biological potential of Tanacetum balsamita L. (Costmary). Plants (Basel).

[40] Wu C, Chen F, Wang X, Wu Y, Dong M, He G, Galyean RD, He L, Huang G (2007). Identification of antioxidant phenolic compounds in feverfew (Tanacetum parthenium) by HPLC-ESI-MS/MS and NMR. Phytochem Anal.

[41] Devrnja N, Krstic-Milosevic D, Janosevic D, Tesevic V, Vinterhalter B, Savic J, Calic D (2021). In vitro cultivation of tansy (Tanacetum vulgare L.): a tool for the production of potent pharmaceutical agents. Protoplasma.

[42] Chen J, Yang J, Ma L, Li J, Shahzad N, Kim CK (2020). Structure-antioxidant activity relationship of methoxy, phenolic hydroxyl, and carboxylic acid groups of phenolic acids. Sci Rep.

[43] Cos P, Rajan P, Vedernikova I, Calomme M, Pieters L, Vlietinck AJ, Augustyns K, Haemers A, Vanden Berghe D (2002). In vitro antioxidant profile of phenolic acid derivatives. Free Radic Res.

[44] Stanciu GD, Luca A, Rusu RN, Bild V, Beschea Chiriac SI, Solcan C, Bild W, Ababei DC (2019). Alzheimer’s disease pharmacotherapy in relation to cholinergic system involvement. Biomolecules.

[45] Patterson C (2018). World alzheimer report 2018, Alzheimer’s Disease International.

[46] Geula C, Darvesh S (2004). Butyrylcholinesterase, cholinergic neurotransmission and the pathology of Alzheimer's disease. Drugs Today (Barc).

[47] Pope C, Karanth S, Liu J (2005). Pharmacology and toxicology of cholinesterase inhibitors: uses and misuses of a common mechanism of action. Environ Toxicol Pharmacol.

[48] Rees TM, Brimijoin S (2003). The role of acetylcholinesterase in the pathogenesis of Alzheimer's disease. Drugs Today (Barc).

[49] Moodie LWK, Sepcic K, Turk T, Frange ZR, Svenson J (2019). Natural cholinesterase inhibitors from marine organisms. Nat Prod Rep.

[50] Uddin MJ, Russo D, Rahman MM, Uddin SB, Halim MA, Zidorn C, Milella L (2021). Anticholinesterase activity of eight medicinal plant species: in vitro and in silico studies in the search for therapeutic agents against Alzheimer’s disease. Evid-Based Complement Altern Med.

[51] Ahmed S, Khan ST, Zargaham MK, Khan AU, Khan S, Hussain A, Uddin J, Khan A, Al-Harrasi A (2021). Potential therapeutic natural products against Alzheimer's disease with reference of Acetylcholinesterase. Biomed Pharmacother.

[52] Loizzo MR, Tundis R, Menichini F (2012). Natural and synthetic Tyrosinase inhibitors as antibrowning agents: an update. Comprehensive Rev Food Sci Food Safety.

[53] Zaidi KU, Ali SA, Ali A, Naaz I (2019). Natural Tyrosinase inhibitors: role of herbals in the treatment of Hyperpigmentary disorders. Mini Rev Med Chem.

[54] Pillaiyar T, Manickam M, Namasivayam V (2017). Skin whitening agents: medicinal chemistry perspective of tyrosinase inhibitors. J Enzyme Inhib Med Chem.

[55] Poovitha S, Parani M (2016). In vitro and in vivo alpha-amylase and alpha-glucosidase inhibiting activities of the protein extracts from two varieties of bitter gourd (Momordica charantia L.). BMC Complement Altern Med.

[56] Gin H, Rigalleau V (2000). Post-prandial hyperglycemia post-prandial hyperglycemia and diabetes. Diabetes Metab.

[57] Lordan S, Smyth TJ, Soler-Vila A, Stanton C, Ross RP (2013). The alpha-amylase and alpha-glucosidase inhibitory effects of Irish seaweed extracts. Food Chem.

[58] Lebovitz HE (1997). alpha-Glucosidase inhibitors. Endocrinol Metab Clin North Am.

[59] van de Laar FA (2008). Alpha-glucosidase inhibitors in the early treatment of type 2 diabetes. Vasc Health Risk Manag.

[60] Etxeberria U, de la Garza AL, Campion J, Martinez JA, Milagro FI (2012). Antidiabetic effects of natural plant extracts via inhibition of carbohydrate hydrolysis enzymes with emphasis on pancreatic alpha amylase. Expert Opin Ther Targets.

[61] Mohamed EA, Siddiqui MJ, Ang LF, Sadikun A, Chan SH, Tan SC, Asmawi MZ, Yam MF (2012). Potent alpha-glucosidase and alpha-amylase inhibitory activities of standardized 50% ethanolic extracts and sinensetin from Orthosiphon stamineus Benth as anti-diabetic mechanism. BMC Complement Altern Med.

[62] Perez-Gutierrez RM, Damian-Guzman M (2012). Meliacinolin: a potent alpha-glucosidase and alpha-amylase inhibitor isolated from Azadirachta indica leaves and in vivo antidiabetic property in streptozotocin-nicotinamide-induced type 2 diabetes in mice. Biol Pharm Bull.

[63] Ali RB, Atangwho IJ, Kuar N, Ahmad M, Mahmud R, Asmawi MZ (2013). In vitro and in vivo effects of standardized extract and fractions of Phaleria macrocarpa fruits pericarp on lead carbohydrate digesting enzymes. BMC Complement Altern Med.

[64] Kim K-T, Rioux L-E, Turgeon SL (2014). Alpha-amylase and alpha-glucosidase inhibition is differentially modulated by fucoidan obtained from Fucus vesiculosus and Ascophyllum nodosum. Phytochemistry.

[65] Durazzo A, D'Addezio L, Camilli E, Piccinelli R, Turrini A, Marletta L, Marconi S, Lucarini M, Lisciani S, Gabrielli P, Gambelli L, Aguzzi A, Sette S (2018). From plant compounds to botanicals and back: a current snapshot. Molecules.

[66] Salehi B, Ata A, Anil Kumar NV, Sharopov F, Ramírez-Alarcón K, Ruiz-Ortega A, Abdulmajid Ayatollahi S, Valere Tsouh Fokou P, Kobarfard F, Amiruddin Zakaria Z (2019). Antidiabetic potential of medicinal plants and their active components. Biomolecules.

[67] Diwan R, Shinde A, Malpathak N. Phytochemical composition and antioxidant potential of Ruta graveolens L. in vitro culture lines. J Bot. 2012;(2012):1–6.

[68] Abdelouhab K, Guemmaz T, Karamac M, Kati DE, Amarowicz R, Arrar L (2023). Phenolic composition and correlation with antioxidant properties of various organic fractions from Hertia cheirifolia extracts. J Pharm Biomed Anal.

[69] Zhang B, Quan H, Cai Y, Han X, Kang H, Lu Y, Cheng H, Xiang N, Lan X, Guo X (2023). Comparative study of browning, phenolic profiles, antioxidant and antiproliferative activities in hot air and vacuum drying of lily (Lilium lancifolium Thunb.) bulbs. LWT.

[70] Daina A, Michielin O, Zoete V (2017). SwissADME: a free web tool to evaluate pharmacokinetics, drug-likeness and medicinal chemistry friendliness of small molecules. Sci Rep.

[71] Mishra S, Dahima R (2019). In vitro ADME studies of TUG-891, a GPR-120 inhibitor using SWISS ADME predictor. J Drug Deliver Ther.

[72] Daina A, Zoete V (2016). A boiled-egg to predict gastrointestinal absorption and brain penetration of small molecules. ChemMedChem.

